# Ion-DNA Interactions as a Key Determinant of Uracil DNA Glycosylase Activity

**DOI:** 10.1021/acs.biochem.5c00067

**Published:** 2025-05-07

**Authors:** Sharon N. Greenwood, Alexis N. Dispensa, Matthew Wang, Justin R. Bauer, Timothy D. Vaden, Zhiwei Liu, Brian P. Weiser

**Affiliations:** † Department of Molecular Biology, Rowan-Virtua School of Osteopathic Medicine, 43987 Rowan University, Stratford, New Jersey 08084, United States; ‡ Department of Molecular Biology, Rowan-Virtua School of Translational Biomedical Engineering & Sciences, 43987 Rowan University, Stratford, New Jersey 08084, United States; § Department of Chemistry & Biochemistry, College of Science and Mathematics, 3536 Rowan University, Glassboro, New Jersey 08028, United States

## Abstract

Because of their ubiquitous presence, ions interact with numerous macromolecules in the cell and affect critical biological processes. Here, we discuss how cations including Mg^2+^ alter the enzymatic activity of a DNA glycosylase by tuning its affinity for DNA. The response of uracil DNA glycosylase (UNG2) to Mg^2+^ ions in solution is biphasic and paradoxical, where low concentrations of the ion stimulate the enzyme, but high concentrations inhibit the enzyme. We analyzed this phenomenon by modeling experimental data with a statistical framework that we empirically derived to understand molecular systems that display biphasic behaviors. Parameters from our statistical model indicate that DNA substrates are nearly saturated with cations under ideal conditions for UNG2 activity. However, the enzyme slows rather abruptly when the ionic content becomes too low or too high due to changes in the electrostatic environment that alter protein affinity for DNA. We discuss how ion occupancy on DNA is dependent on DNA length; thus, the sensitivity of UNG2 to cations is also dependent on DNA length. Finally, we found that Mg^2+^-induced changes in DNA base stacking and dynamics have minimal effects on UNG2, as these outcomes occur at ion concentrations that are much lower than is required for efficient enzyme activity. Altogether, our work demonstrates how cation–DNA interactions, which are likely common in the nucleus, are a key determinant of uracil base excision repair mediated by UNG2.

## Graphical abstract

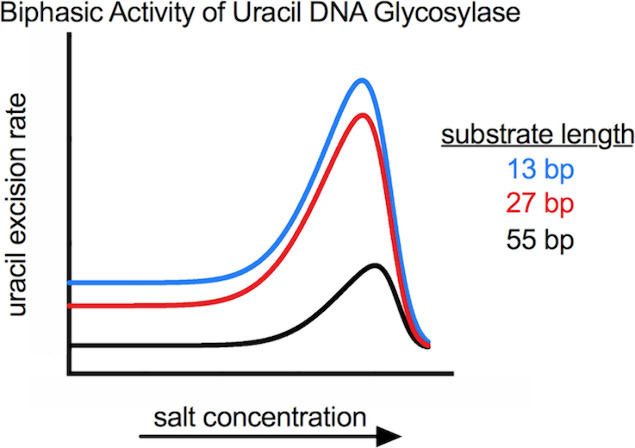

## Introduction

Many have questioned how DNA binding proteins can efficiently scan an entire genome (∼10^9^ bp in humans) to identify specific sequences or base lesions and execute their function. As an example, DNA glycosylases recognize very specific lesions that are rare in the genome, possibly occurring once every ∼10 million nt.^1^ After identifying a damaged base, these enzymes cleave the N-glycosidic bond of the base and release it, producing an abasic site that is successfully repaired to its original sequence with other proteins. DNA glycosylases make multiple electrostatic contacts with the DNA backbone regardless of the sequence or presence of a lesion.^2–6^ This nonspecific binding must be strong enough for the enzyme to remain engaged with DNA until a damaged base is recognized and removed, but the affinity must not be too strong or else the “scanning” speed of the enzyme will slow. DNA glycosylases search DNA through associative transfer (sliding), as well as dissociative transfer where the enzymes “hop” to a distant DNA strand.^7^ Dissociative transfer is especially influenced by the ionic content of the solution because protein binding to DNA releases cations from the DNA ion cloud, which incurs an entropic penalty that is not paid when the enzyme associatively transfers on DNA.^7–9^ As with other DNA binding proteins,^10,11^ DNA glycosylases evolved under physiological salt conditions that should optimize their DNA binding affinities for efficient scanning of DNA and lesion removal.

Much of our understanding on the relationship between ion-DNA interactions and the activity of DNA glycosylases comes from studies with the paradigmatic enzyme uracil DNA glycosylase (UNG2). UNG2 contains a small globular catalytic domain whose biophysical interactions with DNA have been extensively studied in the presence of monovalent cations, which also bind DNA and reduce enzyme-DNA interactions.^8,9,12^ Less is known about how divalent cations such as Mg^2+^ influence the interaction of the enzyme with DNA even though it is often considered a major counterion for DNA in the nucleus. Specific ion binding sites on DNA exist, but they vary depending on the cation and sequence and are not limited to backbone interactions.^13–17^ Divalent cations generally have higher affinity for DNA than monovalent cations, but both affect the structural properties of DNA including its helicity, elasticity, and thermostability.^18–20^ However, given that DNA is a negatively charged polymer that neutralizes with ions following an approximate Poisson–Boltzmann distribution, significant differences between monovalent and divalent cations might not be expected with regards to their effects on protein–DNA interactions. We were therefore surprised by reports that the activity of the UNG2 catalytic domain was relatively insensitive to Mg^2+^ ions,^21–25^ which was in contrast to monovalent cations that disrupt the ability of the enzyme to bind DNA.^8^ Further complicating matters, the full-length UNG2 enzyme was reported to be dramatically stimulated by Mg^2+^.^21–25^ This was reported to occur through an unknown mechanism that required the ∼90 residue N-terminal domain (NTD) of UNG2 which precedes its catalytic domain^21–25^ and is a weak DNA binding domain.^26–29^

In the present work, we aimed to reconcile the disparate reports of how monovalent and divalent cations influence DNA binding and uracil excision activity of UNG2 and its catalytic domain. A consistent finding was that all cations tested (Mg^2+^, Na^+^, and K^+^) had a biphasic effect on the enzyme where low concentrations of ion stimulated its activity, but high concentrations of ion inhibited the enzyme. Such paradoxical systems where low and high doses of an effector produce opposite responses are called “hormetic.” Biphasic/hormetic systems are frequently encountered throughout biology^30^ and enzymology,^22,31–34^ but there is no standard method to statistically model their properties. In our case, we developed a statistical framework to model the biphasic effects of cations on UNG2 activity to extract biochemically relevant parameters from experimental data, such as the ideal ionic environments for enzyme activity. We determined that Mg^2+^ ions influence the activity of UNG2 and its catalytic domain specifically through ion-DNA interactions that affect protein binding affinity for DNA, and this mechanism was consistent with reported effects of monovalent cations on UNG2.^8^ Our findings are important for understanding how ions regulate the activity of critical DNA binding proteins and provides a new statistical approach to model molecular systems yielding biphasic responses.

## Materials and Methods

### Oligonucleotides

All synthetic oligonucleotides were purchased from IDT and purified with denaturing Urea-TBE PAGE in our lab, except for dark quencher-containing oligos, which were purified by IDT using HPLC. Fluorescein end-labels on oligonucleotides were attached to the 5′ end as phosphodiester linkages with a six carbon spacer between the phosphate and fluorescein. The dark quencher that we used was Iowa Black FQ, which was also attached to a 5′ phosphate at the terminal end of the oligonucleotides (the exact chemical structure of the quencher and its linkage was proprietary to IDT). All ssDNA and dsDNA had the same “core” sequence surrounding the uracil base, and U/A bps were used in dsDNA contexts. Where nonspecific DNA was used, the U was substituted with a T or a 2-aminopurine base; every effort was made to maintain the continuity of the DNA sequence on different substrates to eliminate any sequence-dependent variability in the results. All sequences of synthetic DNA used in assays can be found in the Supporting Information.

Generally, oligonucleotides purified with denaturing PAGE were buffer exchanged into 10 mM Tris–Cl (pH 8.0), 100 mM NaCl, and 0.1 mM EDTA which also served as the annealing buffer for preparing duplex DNA. Duplexes were formed by heating complementary oligonucleotides to 95 °C for 5 min then slowly cooling to room temperature. When one of the duplex strands contained a fluorescent 2-aminopurine base or fluorescein label, the unlabeled strand was added during annealing at an 8% excess over the fluorescent strand to ensure that all measured signal derived from duplex DNA. Dark quencher oligonucleotides, which were purified by the manufacturer, were dissolved directly in 10 mM Tris–Cl and 0.1 mM EDTA (pH 8.0) upon receipt.

### Recombinant UNG2 Proteins

Detailed methods for the expression and purification of full-length UNG2 and the UNG2 catalytic domain, which lacks the N-terminal 91 residues of the protein, have been reported previously.^8,26,35,36^ Recombinant UNG2 proteins with point mutations (Q144A and N215A) were produced identically to wild-type UNG2 after standard QuikChange site-directed mutagenesis was performed on the pET21a expression plasmid.^35^ UNG2-Fluor was produced as previously reported.^35^ UNG2-Fluor was a full-length UNG2 protein with its three endogenous cysteines mutated to alanines, and an added N-terminal cysteine that was conjugated to maleimide-fluorescein.^35^ The sequences of all proteins expressed in *E. coli* were verified with Sanger sequencing.

### Uracil Excision Assays

Uracil excision assays were performed at 22 °C in a buffer containing 10 mM Tris–Cl and 0.1 mM EDTA (pH 8.0) that was spiked with different concentrations of MgCl_2_ (0–50 mM), NaCl (0–1 M), or KCl (0–1 M). All of the assays contained an additional ∼2 mM NaCl from diluting our substrate oligonucleotides into the reaction buffer (DNA stocks contained 100 mM NaCl); we show later that this small concentration of NaCl had a negligible effect on UNG2 activity. Because some of our assays were performed in very low ionic solutions (essentially 10 mM Tris–Cl (pH 8.0), 2 mM NaCl, and 0.1 mM EDTA), we used circular dichroism to confirm that dsDNA retained its B-form helical structure under these conditions (Figure S1).

Uracil excision assays contained a final concentration of 0.5 μM DNA, and reactions were initiated by adding UNG2 at a concentration between 0.75 and 4 nM.^26,36,37^ Reactions were quenched with 0.3 M NaOH and heat, which also cleaved abasic sites that resulted from uracil excision.^26,36,37^ For assays containing MgCl_2_, we then added EDTA to a final concentration of 50 mM, which was required to analyze these samples by our standard electrophoresis protocol. Formamide was added to all quenched reactions at a 2.3-fold excess volume, the samples were heated to 95 °C, then the reactions were subjected to denaturing Urea-TBE PAGE.^26,36,37^ Fluorescein end-labels on substrate and product oligonucleotides were imaged in the gels using an Azure c400 imager.^36,37^ The fluorescent substrate and product bands were quantified in FIJI to determine what percent of the substrate was processed.^38^ Then, we calculated the rate of reaction (μM substrate processed per second) and divided this value by the enzyme concentration used in the reaction (in μM) to derive the *k*_obs_ values that we report (in sec^–1^). Because of the vastly different rates for the enzyme in the presence of different ion concentrations, we were not always able to maintain strict steady-state conditions (less than 10–20% substrate processed).

### Biphasic Dose–Response Modeling

Biphasic data sets showing the effects of different salt concentrations on uracil base excision rates generally produced inverted U-shaped curves where the enzyme rate in the absence of salt was detectable and therefore higher than the enzyme rate in the presence of near infinite salt concentrations, which should approach 0. We empirically developed a nonlinear function to model this behavior of UNG2 (the artificial intelligence (AI) platform ChatGPT 4o was used as a tool to facilitate the derivation of eqs 2 and 3).^39^ When the rates (*k*_obs_ values) were plotted on the *y* axis and the salt concentrations were plotted as log_10_(*x*) values, the data sets resembled a skewed or asymmetric Gaussian function. Our model for this data began with a simple symmetric Gaussian function

$$y=A\times \exp\left(-0.5\times {\left(\frac{x-\mu }{\sigma }\right)}^{2}\right)$$ 1

where *A* was the amplitude or the *y* value at the bell-shaped peak, μ was the *x* value at *A* and was analogous to the mean in a Gaussian distribution, and σ was analogous to the standard deviation in a Gaussian distribution (the square root of the variance of *x*) (Figure S2). A *skew* factor was introduced into the equation to allow for an asymmetric shape where the curve was biased to one side, and parameters were renamed to reflect their new roles in the equation

$$y=a\times \exp\left(-0.5\times {\left(\frac{x-m}{s}\right)}^{2}\right)\times (1+\mathrm{erf}(skew\times (x-m)))$$ 2

where the amplitude term *a* scaled the height of the peak, but no longer actually represented the *y* value at the peak; parameter μ was renamed *m* because it no longer represented the true center of the peak, but acted as a reference point for *skew*; and σ no longer represented its usual definition of dispersion, but still governed the width of the distribution, so it was renamed *s*. Finally, the error function modified the shape of the bell curve by amplifying or shrinking one side relative to the other based on the *skew* parameter (Figure S2). In eqs 1 and 2, *y* approaches 0 as log_10_(*x*) approaches negative or positive infinity; however, this did not accurately reflect our data sets where enzyme activity was greater than zero in the absence of salt. Instead, when plotting the enzyme rate *y* at the log_10_ of an infinitesimally low salt concentration, the resulting point should lie on the asymptote at a *y* value equal to the baseline rate in the absence of salt, rather than zero. For this reason, we introduced a logistical sigmoidal function into the equation that allowed independent baselines for the asymptotes:

$$y=a\times \exp\left(-0.5{\left(\frac{x-m}{s}\right)}^{2}\right)\times (1+\mathrm{erf}(skew\times (x-m)))+d+\frac{c-d}{1+\exp(-10\times (x-m))}$$ 3

where *d* was the asymptotic value as log_10_(*x*) approaches negative infinity (enzyme activity in the absence of salt), *c* was the asymptotic value as log_10_(*x*) approaches positive infinity (enzyme activity at very high salt), the sigmoidal steepness parameter was set to 10 to ensure a fast transition between *d* and *c*, and the other parameters retain their definitions from above (Figure S2). Curves were fit to data sets in GraphPad Prism 7 after eq 3 was input as a user-defined equation. Comprehensive tables of curve parameters that were determined for each data set using eq 3 can be found in Tables S1 and S2. Some parameters were constrained to specific values in select data sets to further improve curve fitting (Tables S1 and S2). During modeling, the no salt control (0 mM) data was assigned a concentration of 0.001 or 0.01 mM as seen in the graphs so that it could be plotted on a log_10_ axis.

Accurate curve fitting to our data sets allowed us to extract biologically meaningful parameters that described quantitative features of the biphasic system. Given eq 3 and the curve parameters determined by Prism for each data set (*a*, *c*, *d*, *m*, *s*, and *skew*), we prompted ChatGPT to calculate additional parameters *y*_max_ and *M* by interpolation (ChatGPT used Python code; the interpolated parameter values were then manually confirmed by the user). Parameter *y*_max_ defined the maximum rate of enzyme activity (*k*_obs_ value) and was the *y* value at the top of the stimulatory peak, and parameter *M* was the concentration of salt that produced the maximum enzyme rate or *y*_max_ (Figure 1). The baseline rate of enzyme activity, also known as parameter *d* from eq 3, was the *k*_obs_ for the enzyme in the absence of salt and was the *y* value at the upper asymptote (Figure 1). Fold changes in enzyme activity relative to baseline were calculated as the ratio *y*_max_/*d*.

**Figure 1:**
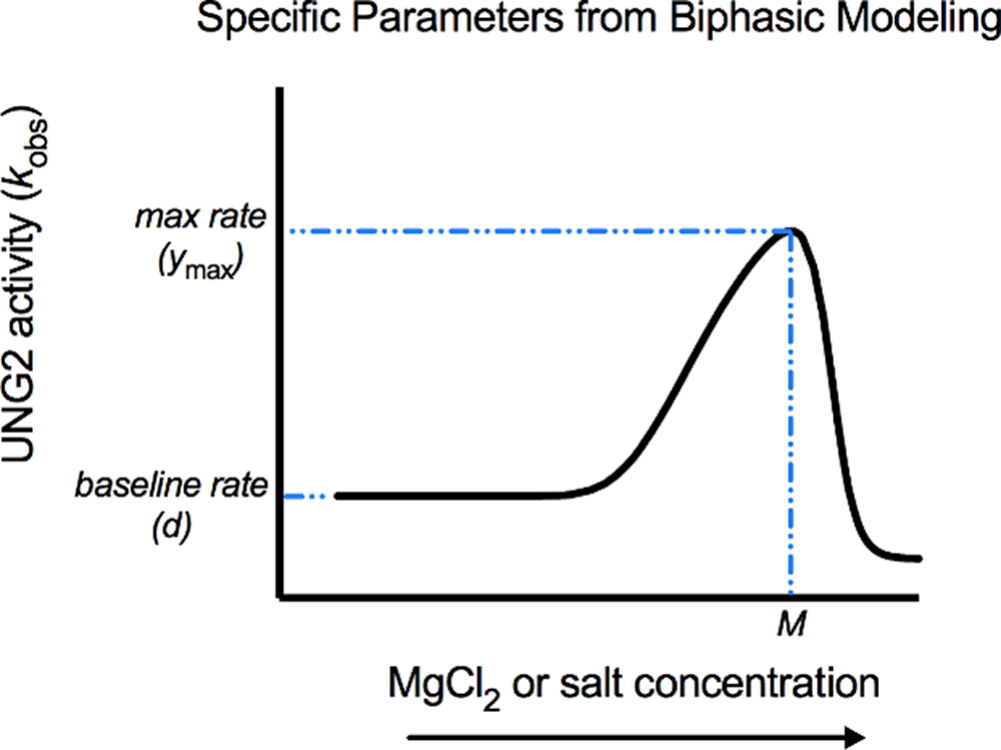
Depiction of specific curve parameters on a biphasic (hormetic) concentration–response curve.

The quality of our hormetic dose–response model was assessed by comparing it to the established Cedergreen model for hormesis^40^ that we previously used for a biochemical data set.^41^ We report a total of 13 biphasic dose–response relationships in this work that we modeled using eq 3 as well as the Cedergreen model:

$$y=c+\frac{(d-c)+f\times \exp\left(\frac{-1}{x^{a}}\right)}{1+\exp\left(b\times \ln\left(\frac{x}{e}\right)\right)}$$ 4

where *a* influenced the rate of increase prior to the hormetic peak, *b* influenced the steepness of the descending part of the curve toward the lower asymptote, *c* was the *y* response value at the lower asymptote as *x* approached infinity, *d* was the *y* response value at the upper asymptote as *x* approached negative infinity, *e* served as a lower bound for the halfway point between *d* and *c*, and *f* influenced the height of the hormetic peak.^40–42^ All 13 data sets could be modeled with eq 3; however, two data sets could not satisfactorily be modeled with eq 4. Of the 11 data sets modeled with both equations, nine of the data sets had improved *R* ^2^_adjusted_ values using eq 3 compared to eq 4 (Figure S2); thus, our model outperformed the Cedergreen equation for these data sets. An extended discussion of modeling our data sets with eq 4 can be found in the Appendix of the Supporting Information.

### Molecular Modeling and Simulation of UNG2

To search for structural evidence of UNG2-Mg^2+^ interactions, we retrieved 98 structures of UNG enzymes from RCSB Protein Data Bank (PDB) by performing a sequence search using amino acid residues 143-270 from the human UNG gene (Uniprot code P13051-1) as input with an E-value cutoff of 0.1. These structures were then searched for the ligand ID “MG” which identified PDB code 5AYR as a structure of the human UNG2 catalytic domain bound to a Mg^2+^ ion and other molecules.^43^ The UNG2 protein chain from 5AYR was aligned to the structure in PDB code 1EMH, which contained human UNG2 bound to pseudouracil-containing DNA, to produce a molecular model of the UNG2 catalytic domain bound to dsDNA with pseudouracil flipped into its active site, a catalytic water molecule adjacent to pseudouracil and Asp145, and a Mg^2+^ ion bound to residues Gln144, Asp145, and Asn215.

To simulate the Mg^2+^ ion bound to the UNG2 catalytic domain, the 5AYR protein structure with bound ion was solvated in a periodic box of explicit OPC water molecules^44^ with the appropriate number of K^+^ and Cl^–^ ions to neutralize the system and reach an ion concentration of 150 mM. The system contained approximately 61,000 atoms. The ff19SB force field was applied to the protein.^45^ The atomistic molecular dynamics simulation was performed using the GPU-accelerated pmemd.cuda engine in Amber22.^46^ The system was equilibrated in a stepwise procedure as described previously,^31^ including solvent minimization, gradual heating to 300 K, and equilibration at 300 K and 1 atm pressure in the NPT ensemble. The production simulation was run for 3 μs in the NVT ensemble at 300 K, employing the particle mesh Ewald method for long-range electrostatics and an 8 Å cutoff for nonbonded interactions.^46^ Trajectory visualization and analysis were performed using VMD^47^ and the CPPTRAJ^48^ module in Amber22, respectively.

### Simulation of Oligonucleotides

To simulate oligonucleotides, ssDNA molecules were built using 3DNA/DSSR.^49,50^ The 7 nt sequence was 5′-CGAUAGC-3′ and the 13 nt sequence was 5′-AATCGAUAGCTAA-3′, which matched sequences used in enzyme assays. The 5′ ends of the in silico uracil-containing oligonucleotides terminated with a 5′ −OH instead of a phosphate. This resulted in an even number of negative charges in the system that could be neutralized with divalent cations alone. The oligonucleotides were entered into CHARMM-GUI’s solution builder and placed in a cubic box before hydrating with TIP3P water molecules.^51–53^ For the 13 nt oligo, the length of the system box was 55 Å, and six Mg^2+^ ions were added to neutralize the system, which contained approximately 15,500 atoms. For the 7 nt oligo, the length of the box was 43.65 Å, and three Mg^2+^ ions were added to neutralize the system, which contained approximately 7,700 atoms. Based on the dimensions of the 13 nt and 7 nt oligo systems and the number of Mg^2+^ ions present, the in silico Mg^2+^ concentration was always 60 mM. The CHARMM36m force field was used,^54^ and simulations were run with NAMD3.0b7.^55^ The simulation stepsize was 2 fs, the pH was 7.4, and the temperature was 310 K. We ran three simulations of the 13 nt oligo (300 ns each) which had different initial water and cation placements. In contrast to the 13 nt oligo systems, we ran a single 1 μs simulation of the shorter 7 nt ssDNA. Interaction of Mg^2+^ ions with DNA was quantified by determining how many ions were within 6 Å of a DNA atom throughout the trajectory; the 6 Å threshold allowed for Mg^2+^ to retain its first hydration shell upon DNA binding.^14^ Simulations were also analyzed with the Clustering plugin on VMD.^56^

### UNG2-Fluor Binding Assays

The interaction of UNG2-Fluor with dark quencher-containing ssDNA was measured in a quartz microcuvette (160 μL) using a Horiba Fluoromax 4 instrument. The temperature was 22 °C, and the buffer contained 10 mM Tris–Cl and 0.1 mM EDTA (pH 8.0) that was spiked with different concentrations of MgCl_2_. To conduct the assay, the dark quencher oligonucleotide was diluted in the cuvette to the desired concentration and its background fluorescence was measured (excitation = 495 nm, emission = 500–700 nm). Then, 1 μL of UNG2-Fluor was added directly to the cuvette to achieve a final concentration of 50 nM, the sample was mixed by pipetting, and the fluorescence was measured again; because UNG2-Fluor was stored in 300 mM NaCl, the assay buffer also contained a final concentration of 2 mM NaCl. The background fluorescence from the dark quencher ssDNA alone was subtracted from the fluorescence that was recorded in the presence of UNG2-Fluor to produce the final emission scans. The fluorescence intensities at 520 nm were extracted from the emission scans for each concentration of dark quencher ssDNA. The fluorescence intensities were plotted against the ssDNA concentrations, and a negative/inverted hyperbolic curve was fit to the data using the quadratic binding equation

$$y=F_{\max}-\left(\frac{F_{\max}-F_{\min}}{2\times L}\right)\times (b-\sqrt{b^{2}-(4\times x\times L)})$$

$$b=K_{d}+x+L$$ 5

where *F*_max_ was the maximum fluorescence intensity of UNG2-Fluor in the absence of dark quencher ssDNA, *F*_min_ was the minimum fluorescence that reflected completely quenched UNG2-Fluor, *L* was the concentration of UNG2-Fluor used in the assays, and *K*_d_ was the dissociation constant for the interaction of UNG2-Fluor with dark quencher ssDNA (note that all of these parameters were estimated from the curves that were fit to the data).

Binding free energies (Δ*G*_bind_) associated with the interaction of UNG2-Fluor and dark quencher ssDNA at different concentrations of MgCl_2_ were calculated using

$$\Delta G_{\mathrm{bind}}=-RT\ln(K_{a})$$ 6

where *R* was the gas constant (1.987 cal K^–1^mol^–1^), *T* was the temperature (295.15 K), and *K*_a_ was the association constant that was calculated from the *K*_d_ values determined above. For discussion on the salt dependence of UNG2-Fluor—ssDNA binding in the context of counterion condensation theory, log_10_-transformed *K*_a_ values for their interaction were plotted as *y* values against the log_10_-transformed MgCl_2_ concentrations at which they were measured as *x* values. The data was fit with the equation for a straight line

$$\log(K_{a})=\log(K_{a}^{0})-N\log[{\mathrm{MgCl}}_{2}]$$ 7

where the slope *N* estimated the number of counterions released from the DNA ion cloud upon protein binding, and *K*_a_^0^ estimated intrinsic binding affinity of UNG2-Fluor and ssDNA in the absence of salt.^57^

### 2-Aminopurine Oligonucleotide Fluorescence Assays

ssDNA or dsDNA containing a 2-aminopurine base was analyzed in a quartz microcuvette (160 μL) at 22 °C in a buffer of 10 mM Tris–Cl and 0.1 mM EDTA (pH 8.0) which was spiked with different concentrations of MgCl_2_. In the experiment, the background fluorescence of the buffer was measured (excitation = 311 nm, emission = 366 nm), then 1 μL of 2-aminopurine—containing DNA was added to a final concentration of 0.5 μM. After mixing by pipetting, the fluorescence was measured again, and the background buffer fluorescence was subtracted from that obtained with DNA to yield the final intensity values. The fluorescence intensity values for the DNA were plotted as *y* values against the MgCl_2_ concentrations as *x* values. We fit a curve to the dsDNA data using eq 5 where *F*_max_ was the maximum fluorescence intensity of the 2-aminopurine duplex in the absence of MgCl_2_, *F*_min_ was the minimum fluorescence that occurred at high MgCl_2_ concentrations, *L* was the concentration of dsDNA used in the assay (0.5 μM), and *K*_d_ was the apparent dissociation constant (*K*_d,apparent_) for the interaction of Mg^2+^ with dsDNA. We fit a curve to the ssDNA data using the quadratic binding equation for a conventional hyperbolic binding isotherm

$$y=F_{\min}-\left(\frac{F_{\min}-F_{\max}}{2\times L}\right)\times \left(b-\sqrt{b^{2}-4\times x\times L}\right)$$

$$b=K_{d}+x+L$$ 8

where *F*_min_ was the minimum fluorescence of 2-aminopurine—containing ssDNA that occurred in the absence of MgCl_2_, *F*_max_ was the maximum fluorescence intensity that occurred at the highest concentrations of MgCl_2_, *L* was the concentration of ssDNA used in the assay (0.5 μM), and *K*_d_ was the apparent dissociation constant (*K*_d,apparent_) for the interaction of Mg^2+^ with ssDNA.^35,58,59^

To calculate the percent occupancy of Mg^2+^ sites that occurred when the activity of UNG2 or its variant was highest for each DNA substrate, we used the equation

$$\%\mathrm{Occupancy}=100\times \frac{10^{\log[M]}}{K_{d,\mathrm{apparent}}+10^{\log[M]}}$$ 9

where parameter *M* was the concentration of MgCl_2_ causing maximal UNG2 activity (Figure 1), and *K*_d,apparent_ described the affinity of Mg^2+^ for sites on ssDNA or dsDNA. This was essentially the basic ligand binding equation

$$\%\mathrm{Occupancy}=100\times \frac{\left[L\right]}{K_{d,\mathrm{apparent}}+[L]}$$ 10

where [*L*] was the concentration of ligand, except we expressed [*M*] logarithmically in eq 9 to graph the results on a log_10_(*x*) axis. Critical for our calculation was that the values for parameter *M* and *K*_d,apparent_ were determined under identical experimental conditions (buffer composition and DNA concentration).

## Results and Discussion

To initially characterize the effects of Mg^2+^ and other ions on UNG2, we performed uracil excision assays using a 55 bp dsDNA substrate with a single U/A bp positioned near the middle of the duplex. The uracil excision activity of the enzyme was very low under low ionic conditions with approximately 1 turnover every 3.5 min (*k*_obs_ = 0.004 s^–1^ in 10 mM Tris–Cl, 0.1 mM EDTA, pH 8.0). UNG2’s ability to remove the uracil base was potently stimulated, then inhibited, by the addition of MgCl_2_ to the buffer in a concentration-dependent manner (Figure 2A). The biphasic curve that we fit to the data determined a 35.8-fold enhancement of enzyme activity that peaked at 10 mM MgCl_2_ (parameter *M* in Table 1). However, UNG2 activity was nearly abolished when 50 mM MgCl_2_ was added to the buffer (Figure 2A).

**Figure 2:**
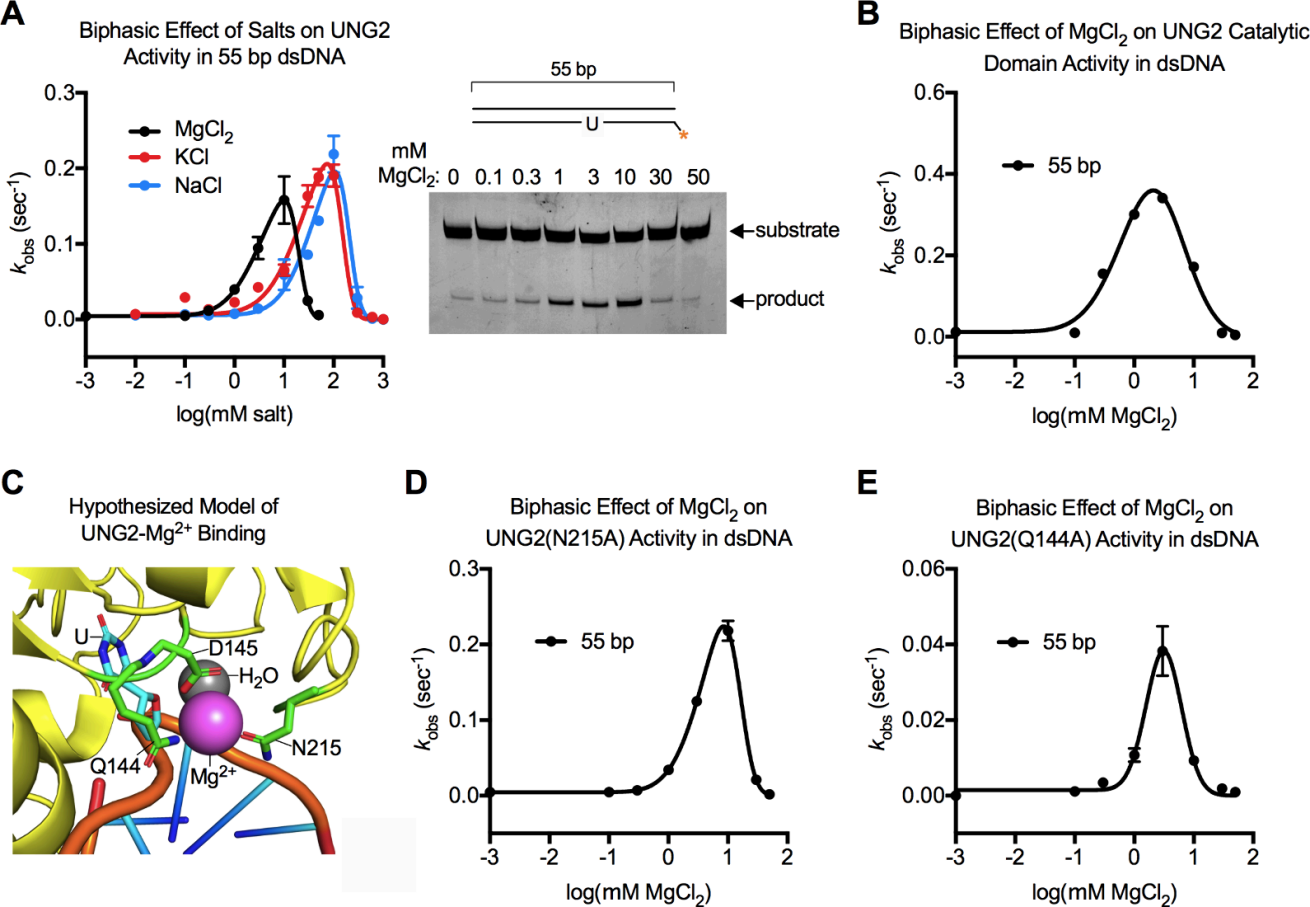
Biphasic effect of MgCl_2_, KCl and NaCl on UNG2 enzymes. (A) Low concentrations of salts stimulated the uracil excision activity of UNG2, while high concentrations of salts inhibited UNG2 activity. A representative Urea-TBE gel from an experiment with MgCl_2_ is shown; gels for KCl, NaCl, and the remaining enzymes in Figure 2 are in Figure S3. (B) Biphasic effect of MgCl_2_ on the UNG2 catalytic domain. (C) Hypothesized (and refuted) model of Mg^2+^ binding to the active site of UNG2. This model combined PDB structures 5AYR, which contained UNG2 bound to Mg^2+^, and 1EMH, which contained UNG2 bound to pseudouracil (U) DNA. (D) Biphasic effect of MgCl_2_ on UNG2­(N215A) activity. (E) Biphasic effect of MgCl_2_ on UNG2­(Q144A) activity.

**Table 1:** Parameters Determined from Modeling the Activity of UNG2 on dsDNA Substrates of Different Lengths

	**assays with MgCl**_**2**_						**KCl**	**NaCl**
	**UNG2**			**catalytic domain**	**UNG2(N215A)**	**UNG2(Q144A)**	**UNG2**	**UNG2**
**parameter**	**13 bp**	**27 bp**	**55 bp**	**55 bp**	**55 bp**	**55 bp**	**55 bp**	**55 bp**
baseline rate (*d*) (s^–1^)	0.1246	0.0805	0.0044	0.012	0.0044	0.0015	0.007	0.0057
maximum rate (*y*_max_) (s^–1^)	0.5124	0.4457	0.1576	0.3595	0.2242	0.0383	0.2061	0.1962
fold-stimulation (*y*_max_/*d*)	4.1	5.5	35.8	30.0	51.0	25.5	29.4	34.4
*M*^a^ (mM)	6.77	6.92	10.04	2.11	8.45	3.13	74.25	106.73

a *M* is the concentration of salt where the maximum rate occurs.

Next, we measured how KCl and NaCl affected the activity of the enzyme. KCl and NaCl enhanced the activity of UNG2 to similar levels as MgCl_2_ (*k*_obs_ = ∼0.2 s^–1^; Figure 2A, Figure S3, and parameter *y*_max_ in Table 1); however, KCl and NaCl were 7–10-fold less potent than MgCl_2_. ∼100 mM of KCl or NaCl was required for peak stimulation of UNG2, and accordingly, even higher concentrations (≥300 mM) significantly reduced UNG2 activity (Figure 2A and Table 1). The difference in potency between MgCl_2_ and the monovalent salts indicated that the Cl^–^ counterion was not a major contributor toward the biphasic activity of the enzyme. In the remaining report, we focused primarily on the molecular basis for the biphasic effects of Mg^2+^ ions on UNG2 because of its potency, although it became apparent that Mg^2+^, K^+^, and Na^+^ may influence the enzyme through a similar mechanism.

### Mg^2+^ Does Not Stimulate UNG2 through Direct Interactions with the Protein

UNG2 contains a ∼90 residue, largely disordered N-terminal domain (NTD) that precedes its catalytic domain,^27,60^ and the NTD was reported to be essential for the stimulation of UNG2 by Mg^2+^ through an unknown mechanism.^21–25^ We challenged these reports by conducting experiments with the purified UNG2 catalytic domain which lacks the first 91 residues of the protein. The catalytic domain was also stimulated and inhibited by MgCl_2_ (Figure 2B). The fit curve determined that the catalytic domain was stimulated 30-fold by 2.1 mM MgCl_2_ (parameter *M* in Table 1). We concluded that the NTD was not required for the stimulation of the enzyme by the ion. However, it was clear that the NTD affected the magnitude of stimulation and the concentration–response of the catalytic domain to Mg^2+^. As discussed later, the NTD is a weak DNA binding domain that is known to affect DNA binding affinity and kinetics of the catalytic domain.^26,27,29,37^

We pursued the possibility that Mg^2+^ might interact directly with a site on the UNG2 catalytic domain to affect the enzyme’s activity. The catalytic domain is highly conserved structurally and functionally across evolution;^61^ however, to our knowledge no members of this protein family were reported to interact with Mg^2+^ or be dependent on the ion for catalysis. We analyzed 98 crystal structures of UNG enzymes for the presence of a Mg^2+^ ion, including 21 structures of the human enzyme alone or bound to various DNA or protein complexes (see Materials and Methods). We identified a single structure of the human catalytic domain bound to a Mg^2+^ ion (PDB code 5AYR) where UNG2 was cocrystallized with a uracil DNA glycosylase inhibitor (UGI) protein from Staphylococcus aureus.^43^ In the crystal structure, a Mg^2+^ ion was coordinated with UNG2 residues Gln144, Asp145, and Asn215 (note that our numbering is according to the PDB structure, not a Uniprot sequence). This was intriguing because Asp145 is in the enzyme active site and initiates catalysis by deprotonating a nucleophilic water that attacks the uracil N-glycosidic bond.^61^ We could model the UNG2 catalytic domain bound to DNA with a pseudouracil base flipped into its active site, the catalytic water adjacent to Asp145, and the Mg^2+^ ion coordinated with the appropriate residues (Figure 2C). We also performed a 3 μs atomistic molecular dynamics simulation with a Mg^2+^-bound catalytic domain as the starting structure derived from 5AYR, and the ion remained stably bound throughout the 3 μs trajectory (Figure S4). Our in silico observations did not consider critical issues such as how Mg^2+^ would dehydrate during protein binding. Nonetheless, there was plausibility for a Mg^2+^ binding site on the UNG2 catalytic domain.

To test whether Mg^2+^ stimulated UNG2 by coordinating with residues Gln144, Asp145, and Asn215, we produced recombinant enzymes with point mutations that would disrupt Mg^2+^ binding (UNG2­(Q144A) and UNG2­(N215A)). The baseline activity of UNG2­(N215A) (parameter *d*) matched wild-type UNG2 (Table 1). UNG2­(N215A) was maximally stimulated 51-fold by 8.5 mM MgCl_2_ (parameter *M*), which was similar to wild-type protein (*M* = 10 mM) (Figure 2D and Table 1). In the absence of MgCl_2_, the baseline activity of UNG2­(Q144A) was virtually undetectable because the side chain of Q144 normally hydrogen bonds with the DNA backbone during catalysis,^62^ and this was disrupted in the mutant. However, even UNG2­(Q144A) was stimulated by MgCl_2_ in a reasonable concentration range (*M* = 3.1 mM) (Figure 2E). Thus, mutation of residues coordinating Mg^2+^ in the catalytic domain of the 5AYR structure had no meaningful effect on the ability of MgCl_2_ to stimulate the enzyme.

These experiments investigated the reasonable evidence that Mg^2+^ interacted directly with the protein to affect its function, which was not supported by our experimental results. We determined that the N-terminal domain of UNG2 was not required for the biphasic effects of Mg^2+^ on the enzyme, and the presence of a Mg^2+^ ion adjacent to the active site in the 5AYR structure was likely an artifact of the crystallization process. We then pursued the possibility that Mg^2+^ influenced UNG2 activity through mechanisms involving direct interactions of the ion with DNA.

### Role of DNA Length in the Stimulation of UNG2 by Mg^2+^

To understand how DNA length affects the stimulation of UNG2 by Mg^2+^, we prepared dsDNA substrates that were 27 bp or 13 bp long and contained a U/A bp in the middle of the duplex; these complemented our initial assays with the 55 bp substrate. In the absence of MgCl_2_, the baseline rate of UNG2 (parameter *d*) increased as the duplex was shortened (Figure 3A and Table 1). This was expected because UNG2 is inhibited by the additional nonspecific DNA present in the longer substrates that hinders its search for the rare uracil base. However, MgCl_2_ enhanced UNG2 activity by only 4.1- or 5.5-fold when the dsDNA substrate was 13 bp or 27 bp, respectively. This was significantly reduced compared to its 35.8-fold stimulation on the 55 bp substrate (Figure 1A). Peak stimulation of the enzyme occurred at a similar concentration of MgCl_2_ for the three duplexes (*M* = 6.8–10 mM) (Table 1).

**Figure 3:**
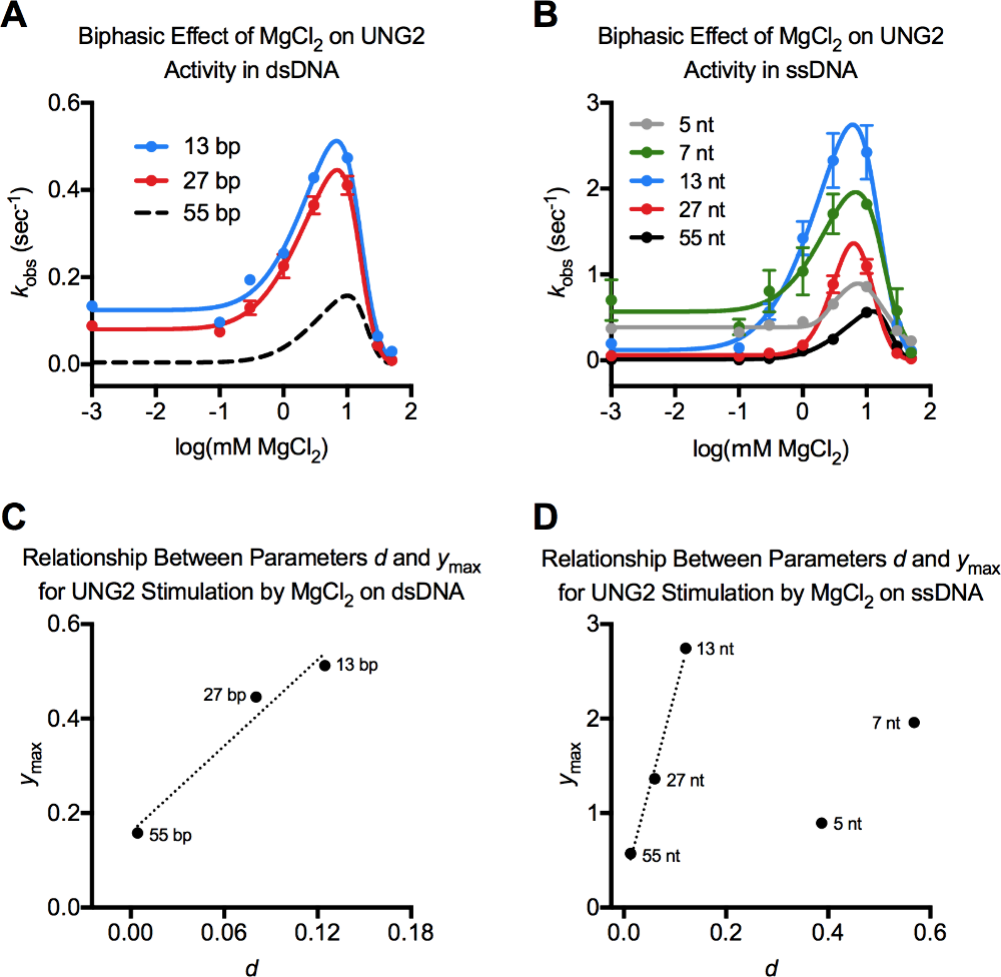
Relevance of DNA length for the biphasic effect of Mg^2+^on UNG2. (A) Effect of MgCl_2_ on the uracil excision activity of UNG2 on dsDNA substrates of different lengths. The 13 and 27 bp substrates had a single U/A bp in the middle of the duplex, and the 55 bp data was presented in Figure 2A. (B) Effect of MgCl_2_ on the uracil excision activity of UNG2 on ssDNA substrates of different lengths. A single uracil base was in the middle of the ssDNA for the 5–27 nt substrates, and 23 nt from the 5′ end of the 55 nt substrate. (C) Correlation between parameter *d* (the uracil excision activity of UNG2 in the absence of salt) and parameter *y*_max_ (the maximum uracil excision activity of UNG2 in the presence of MgCl_2_ at the hormetic peak) for dsDNA substrates shown in panel A. (D) Correlation between parameter *d* and parameter *y*_max_ for ssDNA substrates shown in panel B.

We considered shortening the DNA length further to understand how this variable affects the baseline and maximum rates of the enzyme in our hormetic system. However, cocrystal structures of the catalytic domain with duplex DNA showed that the enzyme only requires a 5 bp segment to establish a productive enzyme–substrate complex,^62^ and duplexes that short were unlikely to remain annealed under our experimental conditions. To simplify our assays, we turned to ssDNA substrates that contained a uracil base to investigate how Mg^2+^ affects UNG2 in single-stranded contexts.

We measured the biphasic effects of Mg^2+^ on the uracil excision activity of UNG2 on ssDNA substrates ranging from 5 to 55 nt long, and again fit curves to the data using eq 3 (Figure 3B and Table 2). As expected, the baseline rate for UNG2 activity in the absence of Mg^2+^ (parameter *d*) generally increased as the ssDNA was shortened because nonspecific DNA was reduced in the assays (Figures 3B and S5). The concentration of Mg^2+^ that maximally stimulated UNG2 was in the same range for each substrate (*M* = 6–12 mM) and was similar to that observed for duplex DNA (*M* = 6.8–10 mM). The maximum rate in the presence of Mg^2+^ (*y*_max_) increased as the ssDNA was shortened from 55 to 13 nt, consistent with the results using duplex DNA; however, a striking result was that the maximum rates from assays using 7 or 5 nt ssDNA were reduced compared to the maximum rate from the 13 nt ssDNA (Figure 3B). This occurred even as the enzyme more efficiently removed uracil from the shorter ssDNA in the absence of Mg^2+^ (see parameter *d*). Thus, the dependence of UNG2 on Mg^2+^ concentration changed when the ssDNA substrate was reduced from 13 to 7 nt.

**Table 2:** Parameters Determined from Modeling the Activity of UNG2 on ssDNA Substrates of Different Lengths

**parameter**	**5 nt**	**7 nt**	**13 nt**	**27 nt**	**55 nt**
baseline rate (*d*) (s^–1^)	0.3872	0.5681	0.1210	0.0603	0.0133
maximum rate (*y*_max_) (s^–1^)	0.8949	1.9591	2.7439	1.3633	0.5718
fold-stimulation (*y*_max_/*d*)	2.3	3.4	22.7	22.6	43.0
*M*^a^ (mM)	7.33	6.73	6.07	6.27	12.37

a *M* is the concentration of MgCl_2_ where the maximum rate occurs.

We used statistical parameters from modeling to verify how DNA length affected the ability of UNG2 to be stimulated by Mg^2+^. Consider how the enzyme rate in the absence of Mg^2+^ (*d*) correlated with the maximum rate that the enzyme could achieve in the presence of ion (*y*_max_) on dsDNA substrates (Figure 3C). A linear relationship between *d* and *y*_max_ will occur when the efficiency of the enzyme in the absence of salt scales proportionally with the extent to which the enzyme can be stimulated by the salt. Linear relationships between *d* and *y*_max_ have been reported in unrelated biological systems producing an array of hormetic responses^63–65^ and indicates that the mechanism causing the biphasic response is stable or consistent as incremental changes are made to the independent variable (in our case, DNA length).^34,41^ In ssDNA substrates, a correlation between *d* and *y*_max_ was also observed for substrates between 13 and 55 nt, but the relationship uncoupled when the ssDNA was shortened to 7 or 5 nt (Figure 3D). Here, a fundamental change occurred in the hormetic system.

To further understand how ion concentration and DNA length jointly affected UNG2 activity, we developed an assay to measure UNG2 interactions with DNA in the absence and presence of salt. We prepared a recombinant UNG2 protein (UNG2-Fluor) that contained an N-terminal fluorescein label and similar activity as the wild-type protein.^35,36^ We equilibrated UNG2-Fluor with a 27 nt ssDNA containing a 5′ dark quencher that dramatically reduced the protein’s fluorescence upon binding (Figure 4A). In the absence of Mg^2+^ or other cations, the interaction of UNG2-Fluor with dark quencher ssDNA was exceptionally strong and superstoichiometric. Using 50 nM of UNG2-Fluor, 83% of its fluorescence was quenched by 10 nM DNA, suggesting at least four protein molecules condensed onto each ssDNA molecule based on their molar stoichiometry (Figure 4B). The addition of MgCl_2_ reduced the binding affinity between UNG2-Fluor and DNA. The *K*_d_ value for their interaction was 3 nM in the presence of 1 mM MgCl_2_, and the *K*_d_ increased to 11 μM with 50 mM MgCl_2_ (Figure 4C).

**Figure 4:**
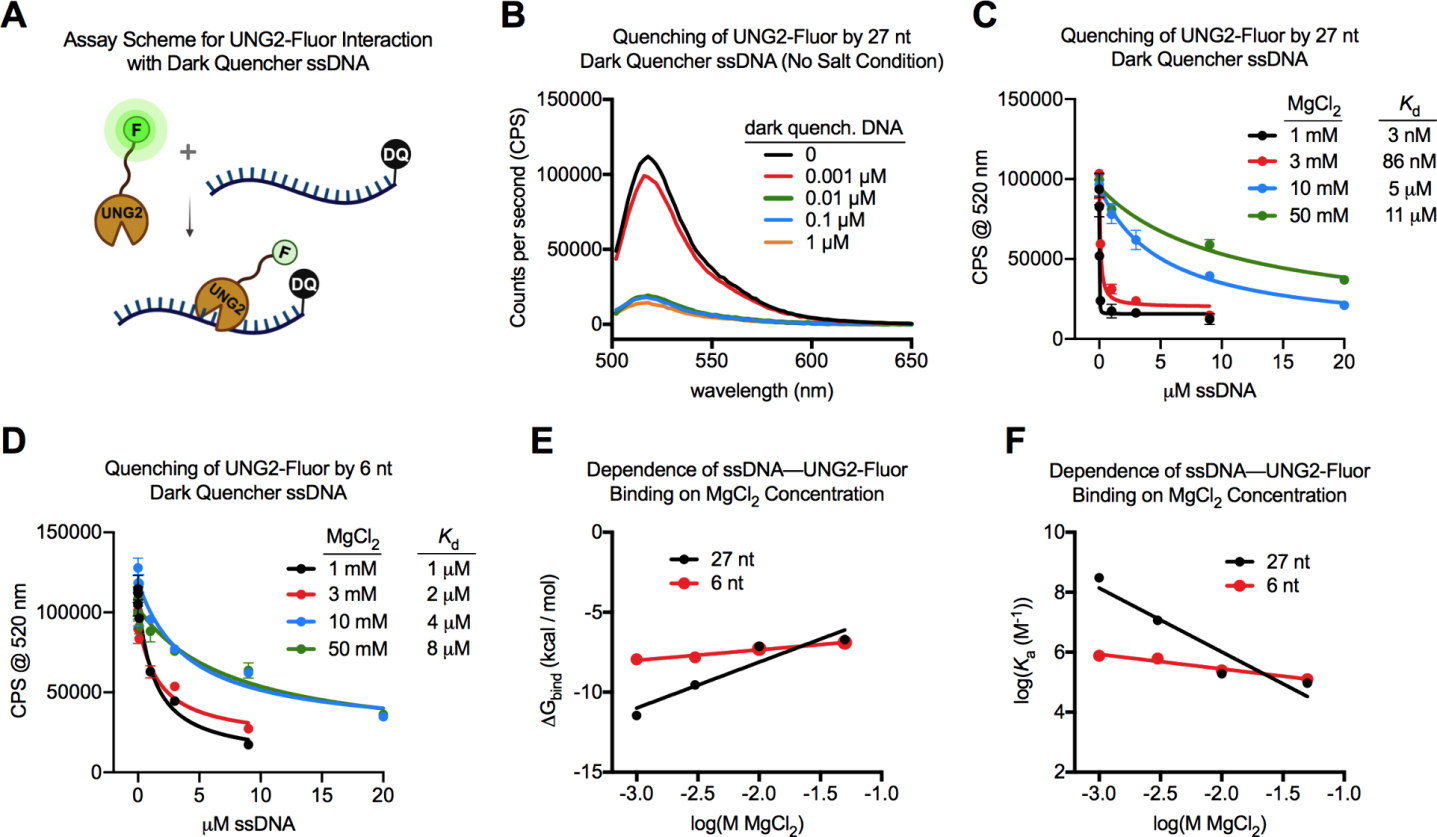
Relevance of MgCl_2_ concentration on the binding affinity of UNG2-Fluor for ssDNA. (A) Schematic of binding assay where the fluorescence intensity of UNG2-Fluor, which contains an N-terminal fluorescein, becomes quenched upon binding a ssDNA that contains a 5′ dark quencher. (B) Fluorescence emission spectra of 50 nM UNG2-Fluor equilibrated with the indicated concentrations of dark quencher ssDNA in a buffer containing 10 mM Tris–Cl and 0.1 mM EDTA (pH 8.0). The excitation wavelength was 495 nm, and error bars from the triplicate measurements were omitted for clarity. (C) Binding isotherms showing quenching of UNG2-Fluor fluorescence by increasing amounts of 27 nt dark quencher ssDNA in the presence of different MgCl_2_ concentrations. The excitation wavelength was 495 nm, and the emission at 520 nm was plotted in the graph. (D) Binding isotherms showing quenching of UNG2-Fluor fluorescence by increasing amounts of 6 nt dark quencher ssDNA in the presence of different MgCl_2_ concentrations. The excitation wavelength was 495 nm, and the emission at 520 nm was plotted. (E) Binding free energies for the ssDNA—UNG2-Fluor interaction were plotted against the MgCl_2_ concentration at which they were measured. The slope for the 27 nt ssDNA data (2.9 ± 0.7) was significantly steeper than the 6 nt ssDNA data (0.7 ± 0.1) indicating that the protein binding affinity for the longer oligonucleotide had a stronger dependence on ionic environment. (F) Binding affinities at different MgCl_2_ concentrations were analyzed in the context of counterion condensation theory. The absolute value of the slopes estimated the number of Mg^2+^ ions released from the DNA ion cloud upon protein binding (2.1 ± 0.5 for the 27 nt ssDNA and 0.5 ± 0.1 for the 6 nt ssDNA). Values in this figure legend were represented as mean ± SE.

The ssDNA/UNG2-Fluor binding assay was repeated with a 6 nt dark quencher oligo that revealed much weaker binding in the absence of salt between the enzyme and short substrate. The *K*_d_ for the interaction of UNG2-Fluor with the 6 nt ssDNA was 1 μM in the presence of 1 mM MgCl_2_ (Figure 4D), which was 2 orders of magnitude weaker than the enzyme’s affinity for the 27 nt ssDNA under the same conditions. The *K*_d_ reduced to 8 μM when UNG2-Fluor interacted with the 6 nt ssDNA in 50 mM MgCl_2_. The binding free energy (Δ*G*_bind_) was much less dependent on salt concentration for the 6 nt ssDNA compared to the 27 nt ssDNA (Figure 4E). We also analyzed the salt dependence of the UNG2-ssDNA binding affinities in the context of counterion condensation theory.^8,57,66,67^ By plotting log­(*K*_a_) from binding assays against log­[MgCl_2_], the slope estimated the number of counterions released from DNA upon protein binding (eq 7). This method estimated that 2.1 Mg^2+^ ions were released from 27 nt ssDNA upon UNG2-Fluor binding, compared to only ∼0.5 Mg^2+^ ions released from the 6 nt ssDNA (in other words, 0 or 1 ions would be released on average) (Figure 4F). The 6 nt ssDNA was unlikely to behave like a polyelectrolyte and hold uniform counterion condensation,^17^ violating assumptions of counterion condensation theory; thus, we advise caution in interpreting the slope and extrapolated parameters for the short oligo (such as the *y*-intercept). Nonetheless, it was plausible that two Mg^2+^ ions released from the 27 nt ssDNA upon UNG2 binding, but not from the 6 nt ssDNA.

To test computationally how many ions would bind a short oligonucleotide, we simulated a 7 nt oligo for 1 μs which averaged only 0.9 Mg^2+^ ions bound at a given time (the oligo contained a 5′ −OH and six phosphates on its backbone). This was consistent with our findings above that UNG2-Fluor displaced 1 ion or less from the 6 nt dark quencher ssDNA. For comparison, we performed three simulations of a 13 nt uracil-containing oligo (300 ns each) and measured 2.7 ± 0.2 Mg^2+^ ions bound to the ssDNA at a given time (mean ± SE). In all cases, the simulated oligonucleotides sampled transient, heterogeneous structures (Figure S6).

Why did UNG2 have significantly higher affinity for 27 nt ssDNA compared to 6 nt ssDNA in the absence of salt? Even though 6 nt is long enough to accommodate the UNG2 catalytic domain, this ssDNA is not long enough to simultaneously interact with the NTD of UNG2.^26,27^ Electrostatics drive DNA interactions with both the catalytic domain and NTD, and a favorable enthalpic gain is expected when both domains engage a longer ssDNA, especially under low salt conditions.^26^ We concluded that the binding mode of UNG2 fundamentally changed in activity assays when the ssDNA was shortened from 13 to 7 nt. Additionally, it was likely that Mg^2+^ counterions were less uniform on 5–7 nt substrates compared to longer DNA due to reduced phosphate charge density and weaker electrostatic field, further altering the dependence of enzyme binding on salt concentration. Both of these factors contributed to the uncoupling of the hormetic system that we observed by altering the efficiency of the enzyme on short ssDNA substrates compared to longer substrates (Figure 3D).

### Affinity of Mg^2+^ for DNA Sites and Effects of Mg^2+^ on Base Stacking

We conducted experiments with ssDNA or dsDNA that contained the synthetic base 2-aminopurine because its fluorescence intensity is sensitive to changes in stacking interactions,^68–70^ which are known to be affected by cations.^71,72^ For dsDNA, we prepared a 13 bp duplex with a uracil—2-aminopurine bp in the center, but the DNA otherwise the same sequence as our other oligonucleotides. MgCl_2_ reduced the fluorescence of 2-aminopurine with a *K*_d,apparent_ of 0.4 mM (Figure 5A). The *K*_d,apparent_ defined the concentration of ion required to half-saturate its sites on the duplex, which was an approximation of Mg^2+^ affinity for DNA. In this assay, the 2-aminopurine base became more stacked in dsDNA as MgCl_2_ was added and interbase quenching of 2-aminopurine was promoted.^68–70^ Others have also reported that low levels of Mg^2+^ and other cations enhance base stacking in dsDNA through ion-phosphate interactions.^73,74^

**Figure 5:**
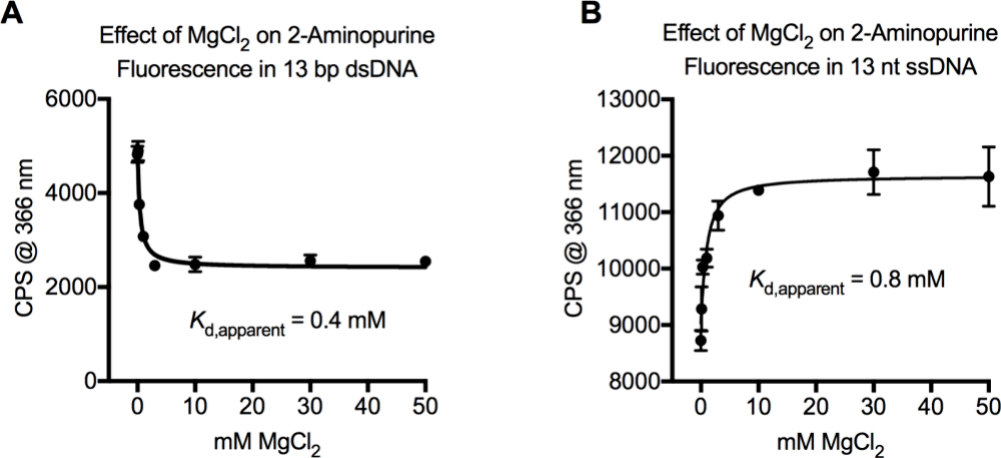
Affinity of Mg^2+^ for DNA sites and effects of Mg^2+^ on base stacking. (A) MgCl_2_ reduced the fluorescence of a 2-aminopurine base positioned in the middle of a 13 bp duplex indicating an enhancement of base stacking caused by Mg^2+^-DNA interactions. The 2-aminopurine was paired with a uracil base. The *K*_d,apparent_ is an approximation of the binding affinity between Mg^2+^ and its sites on dsDNA. (B) MgCl_2_ increased the fluorescence of a 2-aminopurine base positioned in the middle of a 13 nt ssDNA indicating a small reduction in base stacking. The *K*_d,apparent_ is an approximation of the binding affinity between Mg^2+^ and its sites on ssDNA.

To observe how Mg^2+^ affected base dynamics in ssDNA, we prepared a 13 nt ssDNA that had a 2-aminopurine base in the middle of the strand. As MgCl_2_ was added to the buffer, the fluorescence of 2-aminopurine increased then saturated in a traditional binding isotherm (Figure 5B). We determined a *K*_d,apparent_ of 0.8 mM for the interaction of Mg^2+^ with ssDNA. The increased fluorescence in the presence of MgCl_2_ suggested that the 2-aminopurine base became more dynamic and less stacked with other bases in its transient ssDNA structures.^68–72^ However, the overall change in 2-aminopurine fluorescence induced by MgCl_2_ was quantitatively small for both ssDNA and dsDNA (less than 2-fold). 3- to 8-fold changes in 2-aminopurine fluorescence occur when the base transitions fully between stacked and unstacked conformations.^37,68^ We concluded that Mg^2+^ binding to DNA had small but measurable effects on base stacking and dynamics.

### Mechanism for the Biphasic (Hormetic) Effect of Mg^2+^ Ions on UNG2 Activity

Our experimental results support the following mechanism for the biphasic response of UNG2 activity to increasing cation concentrations. UNG2 has very high affinity for DNA in the absence of Mg^2+^ or other cations. The strong electrostatic interactions between the enzyme and DNA backbone likely limit the enzyme’s ability to search bulk DNA for rare uracil bases and limits turnover. Moderate amounts of salt enhance the enzyme’s kinetics by reducing its affinity for nonspecific DNA and allowing it to efficiently search DNA for uracil. However, progressively higher cation concentrations continue to weaken the association of UNG2 with DNA by competing for the negatively charged DNA backbone. Supporting these observations are experiments showing that monovalent cations reduce the affinity of UNG2’s catalytic domain for DNA in a monophasic manner by slowing the enzyme’s on-rate for DNA binding.^8^ The NTD of UNG2 affects the salt sensitivity of the enzyme because it is a weak DNA binding domain driven by electrostatics,^26,27^ but the NTD is not required for a biphasic salt dependence.

We find that the activity of UNG2 is affected by Mg^2+^ solely because the ion interacts with DNA and not the protein. We determined apparent affinities for Mg^2+^ interacting with ssDNA (*K*_d,apparent_ = 0.8 mM) and dsDNA (*K*_d,apparent_ = 0.4 mM). The *K*_d,apparent_ values were much stronger than parameter *M*, which defined the ion concentration at which UNG2 was maximally stimulated by Mg^2+^ (*M* = 6–12 mM for ssDNA and *M* = 2–10 mM for dsDNA). This suggested very high occupancy of available Mg^2+^ sites under conditions where UNG2 activity was optimal. Using eq 9, we calculated the occupancy of Mg^2+^ sites at parameter *M* for each enzyme/substrate pair in Tables 1 and 2. UNG2 and its variants thrived under conditions where ion binding sites were largely occupied with minimal excess ion in solution (Figure 6A,B). UNG2 activity declined when high solution concentrations of Mg^2+^ provided greater likelihood that ions would compete for DNA binding or screen phosphate charge. The entropic view was that the Mg^2+^ gradient that existed between the DNA ion cloud and the bulk solvent reduced at high ion concentrations, and therefore ion release from DNA upon protein binding was less favorable (i.e., the cratic entropy of mixing became less favorable).

**Figure 6:**
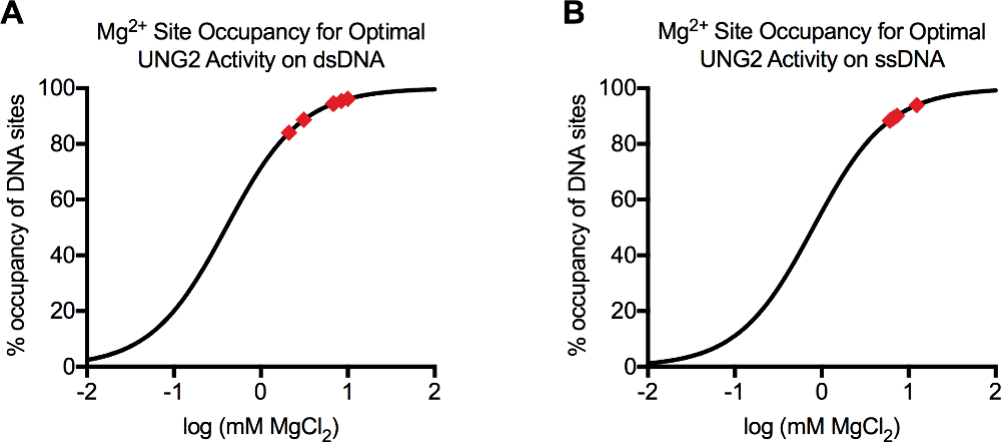
Occupancy of Mg^2+^ sites on DNA in optimal ionic conditions for UNG2 and its variants. (A) Maximum activity of UNG2 and its variants on dsDNA occurred when Mg^2+^ sites were 84–95% occupied. Calculated site occupancies were shown as red diamonds. These were determined using eq 9 which utilized the *K*_d,apparent_ of Mg^2+^ for dsDNA and the concentration of MgCl_2_ producing maximal UNG2 activity for each enzyme/dsDNA pair (parameter *M* values in Table 1). (B) Maximum activity of UNG2 on ssDNA occurred when Mg^2+^ sites were 88–94% occupied. These were determined using eq 9 which utilized the *K*_d,apparent_ of Mg^2+^ for ssDNA and parameter *M* values in Table 2.

Finally, Mg^2+^-induced changes to DNA base stacking may have contributed slightly to the kinetics of UNG2, but was unlikely to significantly contribute to the hormetic mechanism. Mg^2+^ affected base stacking at ion concentrations significantly lower than parameter *M*. The enzyme is also known to prefer bases that are loosely stacked or extrahelical,^3,75^ which is contradictory to the pro-stacking effect that Mg^2+^ had on uracil-containing dsDNA (Figure 5A). Mg^2+^ and other cations can alter DNA structure, dynamics, and mechanical properties in additional ways that were not explored here,^14,18–20^ which could further tune the affinity of UNG2 for DNA and alter its kinetics in the concentration range of ions where hormesis occurs.

### Discussion on Biphasic Statistical Modeling

The statistical model that we developed (eq 3) was empirically derived to fit nonlinear curves to our biphasic data sets, and subsequently, we calculated *y*_max_ and *M* from the curves by interpolation. Our empirical approach contrasted theory-driven models where each parameter defines a specific physicochemical feature of a biochemical system.^76–79^ The parameters in eq 3 are interdependent, i.e., changing one parameter can force an adjustment in another to achieve a good fit. This provides flexibility to the model which might be applied to many different biphasic systems. However, our model was originally designed for bell-shaped data sets where the height of the curve was high compared to the difference between the baselines. This may be an important feature of the data for the Gaussian component of the equation to dominate over the sigmoidal component. Some parameters have straightforward meaning: *d* was the baseline activity of UNG2 in the absence of salt, and *c* was its theoretical activity at infinite salt concentrations (zero). Other parameters describe intuitive features of a hormetic curve such as the orientation of the peak (inverted U-shaped or U-shaped, controlled by the sign of *a*), the width of the peak (controlled by the magnitude of *s*), the position of the peak along the *x* axis (controlled by *m*), or the degree and direction of the peak’s asymmetry (controlled by the magnitude and sign of *skew*). Equation 3 can be reparameterized to explicitly contain variables like *y*_max_ or *ED*_50_,^41,42,80^ thus adding more direct physicochemical meaning to the parameters, but we found this unnecessary with the ability of AI to assist with calculations that define specific points on the curves. Additional parameters describing the biphasic system can be determined from curves fit with eq 3 such as the steepness of the ascending and descending slopes (calculated as the first derivatives at the inflection points), which reflect how sensitive the system is to incremental changes in the *x* variable, and the concentration range where *x* produced a stimulatory response that was higher than or equal to the baseline.^64^ This should be considered when using eq 3 on other biological data sets.

## Conclusions

We used enzymology and statistical modeling to characterize how ion-DNA interactions are a key determinant for UNG2 activity on both ssDNA and dsDNA. Low concentrations of cations including Mg^2+^ stimulated the enzyme, but high concentrations inhibited its activity. The activities of other DNA glycosylases have similar biphasic responses to Mg^2+^ which may arise from a similar mechanism or have different concentration dependences.^21,81^ Even though we focused on Mg^2+^ effects, it is possible that other cations in the nucleus also tune enzyme activity through DNA interactions, and that the concentration response of UNG2 to cations depends on the mixture of ions or other macromolecules neutralizing DNA at a given moment.^8,20,23^ Our research was bolstered by interpreting an enzymatic mechanism in the context of statistical parameters. Our modeling of real data was strengthened by AI, which performed well in mathematical and coding tasks. We intend to use our general approach combining biochemistry and statistical modeling to understand quantitative properties and underlying mechanisms of other molecular systems that exhibit biphasic responses.

## Supplementary Material



## Data Availability

The experimental data sets underlying this study are openly available on zenodo at https://zenodo.org/records/14796319 or DOI: 10.5281/zenodo.14796319.

## Notes

Sequences of oligonucleotides used in this work; circular dichroism spectrum of B-form DNA in low ionic conditions; derivation of eq 3; parameters from modeling UNG2 activity on dsDNA using eq 3; parameters from modeling UNG2 activity on ssDNA using eq 3; representative gels showing biphasic effects of ions on UNG2 proteins; simulation of UNG2 catalytic domain bound to a Mg^2+^ ion; relationship between UNG2 activity in the absence of salt and ssDNA length; structural clusters of 13 nt ssDNA and 7 nt ssDNA simulated with Mg^2+^ ions; Appendix: modeling biphasic data sets with the Cedergreen equation (PDF)

## Notes

UNG2, P13051-1
