# Supplementary material for: Ion-DNA Interactions as a Key Determinant of Uracil DNA Glycosylase Activity
Source: Biochemistry. 2025 May 7;64(10):2332–44. doi: 10.1021/acs.biochem.5c00067 (PMC12096439; doi:10.1021/acs.biochem.5c00067)
Supplement: Supplementary file 1 [file bi5c00067_si_001.pdf]

Supporting Information

Ion-DNA Interactions as a Key Determinant of Uracil DNA Glycosylase Activity

Sharon N. Greenwood,[1,2] Alexis N. Dispensa,[1,2] Matthew Wang,[1,2] Justin R. Bauer,[1,2] Timothy D. Vaden,[3] Zhiwei Liu,[3] Brian P. Weiser[1,2,*]

[1]Department of Molecular Biology, Rowan-Virtua School of Osteopathic Medicine, Rowan University, Stratford, New Jersey 08084, United States of America

[2]Department of Molecular Biology, Rowan-Virtua School of Translational Biomedical Engineering & Sciences, Rowan University, Stratford, New Jersey 08084, United States of America

[3]Department of Chemistry & Biochemistry, College of Science and Mathematics, Rowan University, Glassboro, New Jersey 08028, United States of America

*corresponding author: weiser@rowan.edu (email)

Sequences of oligonucleotides used in this work.

(*i*) 55 bp dsDNA substrate containing a single uracil base near the middle

For: 5′-ACTTAGCTATCGATTCCCGCATGCTCTTAGCTATCGATTCACCTGAGACTAGT CA

Rev: 5′-fluorescein-TGACTAGTCTCAGGTGAATCGAUAGCTAAGAGCATGCGGGAATCG ATAGCTAAGT

(*ii*) 27 bp dsDNA substrate containing a single uracil base in the middle

For: 5′-CATGCTCTTAGCTATCGATTCACCTGA

Rev: 5′-fluorescein-TCAGGTGAATCGAUAGCTAAGAGCATG

(*iii*) 13 bp dsDNA substrate containing a single uracil base in the middle

For: 5′-TTAGCTATCGATT

Rev: 5′-fluorescein- AATCGAUAGCTAA

(*iv*) 55 nt ssDNA substrate containing a single uracil base near the middle

5′-fluorescein-TGACTAGTCTCAGGTGAATCGAUAGCTAAGAGCATGCGGGAATCGATA GCTAAGT

(*v*) 27 nt ssDNA substrate containing a single uracil base in the middle

5′-fluorescein-TCAGGTGAATCGAUAGCTAAGAGCATG

(*vi*) 13 nt ssDNA substrate containing a single uracil base in the middle

5′-fluorescein-AATCGAUAGCTAA

(*vii*) 7 nt ssDNA substrate containing a single uracil base in the middle

5′-fluorescein-CGAUAGC

(*viii*) 5 nt ssDNA substrate containing a single uracil base in the middle

5′-fluorescein-GAUAG

(*ix*) 27 nt ssDNA containing a dark quencher for binding assays

5′-Iowa Black (FQ)-TCAGGTGAATCGATAGCTAAGAGCATG

(*x*) 6 nt ssDNA containing a dark quencher for binding assays

5′-Iowa Black (FQ)-CGATAG

(*xi*) 13 nt ssDNA containing a single 2-aminopurine base (P) in the middle

5′-AATCGAPAGCTAA

(*xii*) 13 bp dsDNA containing a single 2-aminopurine base (P) in the middle paired with uracil

For: 5′-TTAGCTPTCGATT

Rev: 5′-AATCGAUAGCTAA

(*xiii*) 17 bp dsDNA containing a single uracil in the middle and analyzed with circular dichroism

For: 5′-TCTTAGCTATCGATTCA

Rev: 5′-TGAATCGAUAGCTAAGA

Figure S1. Circular dichroism spectrum showing helical B-form DNA in low ionic conditions. The circular dichroism spectra of a 17 bp dsDNA containing a U/A bp in the middle is shown. The duplex was annealed in standard salt conditions (100 mM NaCl) then diluted into a very low ionic buffer (final = 1.6 mM NaCl) for spectroscopy. The oligonucleotide retained classic B-form structure with a negative band at 245 nm and a positive band at 280 nm. The methodology is beneath the figure.

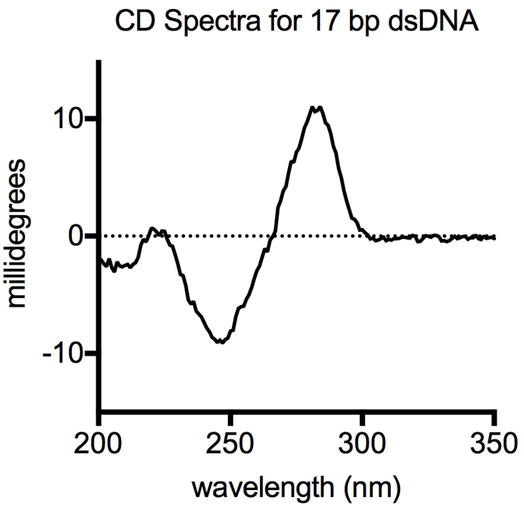

Spectroscopy was performed with a Jasco J-1500CD instrument with a Peltier temperature controller set to 25°C.[1,2] A 1 cm quartz cuvette was used with a sample volume of 1500 µl. Oligonucleotides for the duplex were annealed in 10 mM Tris-Cl (pH 8.0), 100 mM NaCl, and 0.1 mM EDTA then diluted 1/62 to achieve a final buffer of 10 mM Tris-Cl (pH 8.0), 1.6 mM NaCl, and 0.1 mM EDTA. The final DNA concentration was 5 µM.

Figure S2. Derivation of the biphasic (hormetic) dose-response equation 3. (A) A curve was fit using a simple symmetric gaussian function (eq. 1 in the main text) to the biphasic dataset showing the effects of KCl on UNG2 activity in dsDNA. (B) This curve was fit to the same dataset using eq. 2 which asymmetrically skewed the gaussian function. (C) This curve was fit using eq. 3 which introduced independent baselines for the asymptotes into the equation. This curve was also shown in the main text (Figure 2A). (D) Comparison of $R^2_{adjusted}$ values from curves fit to thirteen datasets derived from this work using two different equations. Eq. 3 was developed in this work, and eq. 4 was the Cedergreen model (this is also eq. A1 in the Supporting Information Appendix).[3,4] Two datasets shown as red diamonds could not be satisfactorily modeled with eq. 4, but were modeled with eq. 3; these datasets were from the effects of KCl and NaCl on the biphasic activity of UNG2. Of the eleven datasets that could be modeled with both equations, nine had improved $R^2_{adjusted}$ values using eq. 3 compared to eq. 4.

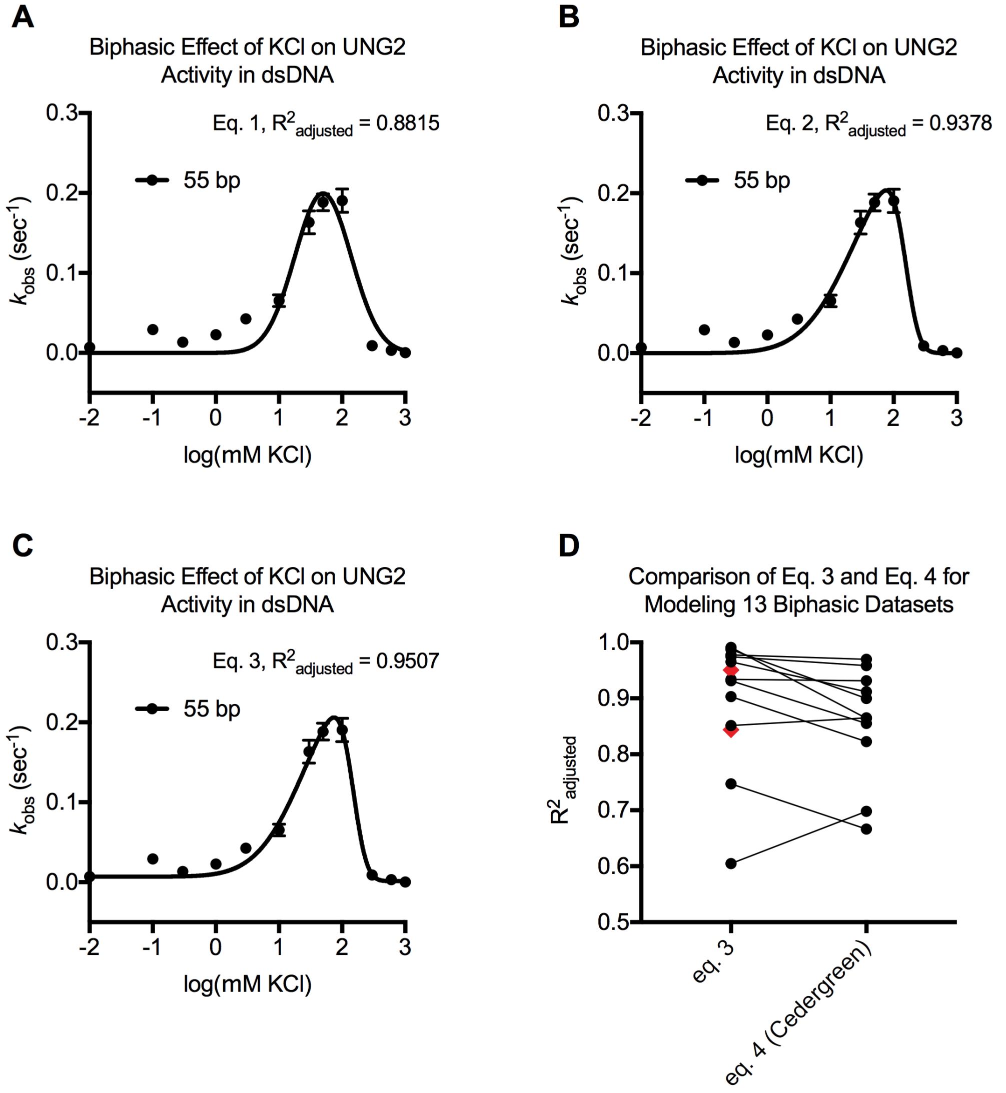

Table S1. Parameters from Modeling the Activity of UNG2 on dsDNA Substrates of Different Lengths Using Equation 3 in the Main Text

	Assays with $MgCl_2$						KCl	NaCl
	UNG2			Catalytic domain	UNG2(N215A)	UNG2(Q144A)	UNG2	UNG2
**Parameter**	**13 bp**	**27 bp**	**55 bp**	**55 bp**	**55 bp**	**55 bp**	**55 bp**	**55 bp**
a	0.2355	0.2129	0.0872	0.2759	0.1348	0.0351	0.1124	0.1100
c	0[a]	0[a]	0[a]	0[a]	0[a]	0[a]	0.001	-0.0035
d	0.1246	0.0805	0.0044	0.012[b]	0.0044	0.0015	0.007[b]	0.0057[b]
m	1.208	1.199	1.297	0.6945	1.204	0.6043	2.173	2.321
s	0.7780	0.8111	0.7192	0.7046	0.5733	0.3191	0.7562	0.6839
skew	-2.804	-3.272	-4.106	-1.133	-3.538	-1[c]	-4.112	-4[c]
$R^2_{adjusted}$	0.9775	0.9338	0.7474	0.9745	0.9874	0.9033	0.9507	0.8438
**Parameters Below Were Determined by Interpolation**								
y_{max} **(sec^{-1})**	0.5124	0.4457	0.1576	0.3595	0.2242	0.0383	0.2061	0.1962
**fold-stimulation (y_{max} / *d*)**	4.1	5.5	35.8	30.0	51.0	25.5	29.4	34.4
M **(mM)**	6.77	6.92	10.04	2.11	8.45	3.13	74.25	106.73

[a]Parameter was fixed during modeling and represents the theoretical activity of UNG2 in the presence of infinite salt concentrations (~zero activity).

[b]Parameter was fixed during modeling. Value was the mean k_{obs} that we measured for the enzyme in the absence of salt.

[c]Parameter was fixed during modeling. Value was chosen to center the curve on the peak data point.

Table S2. Parameters from Modeling the Activity of UNG2 on ssDNA Substrates of Different Lengths Using Equation 3 in the Main Text

Parameter	5 nt	7 nt	13 nt	27 nt	55 nt
a	0.5684	0.9227	1.571	0.9623	0.322
c	0[a]	0[a]	0[a]	0[a]	0[a]
d	0.3872	0.5681	0.1210	0.0603	0.0133
m	0.5901	1.249	1.186	1.04	1.37
s	0.5952	0.7774	0.8681	0.4411	0.6531
skew	2.699	-1.957	-2.596	-2.356	-4.202
$R^2_{adjusted}$	0.9315	0.6046	0.8514	0.9651	0.9912
**Parameters Below Were Determined by Interpolation**					
y_{max} **(sec^{-1})**	0.8949	1.9591	2.7439	1.3633	0.5718
**fold-stimulation (y_{max} / *d*)**	2.3	3.4	22.7	22.6	43.0
***M* (mM)**	7.33	6.73	6.07	6.27	12.37

[a]Parameter was fixed during modeling and represents the theoretical activity of UNG2 in the presence of infinite salt concentrations (~zero activity).

Figure S3. Biphasic effect of $MgCl_2$, KCl, and NaCl on UNG2 enzymes—Representative gels related to Figure 2 of the main text. (A) Biphasic effect of KCl on UNG2 activity. Low concentrations of KCl stimulated the uracil excision activity of UNG2, while high concentrations inhibited UNG2 activity, as shown with a Urea-TBE gel. (B) Biphasic effect of NaCl on UNG2 activity. (C) Biphasic effect of $MgCl_2$ on the UNG2 catalytic domain. (D) Biphasic effect of $MgCl_2$ on UNG2(N215A) activity. (E) Biphasic effect of $MgCl_2$ on UNG2(Q144A) activity. Because of difficulty viewing faint bands, we showed triplicate reactions for UNG2(Q144A).

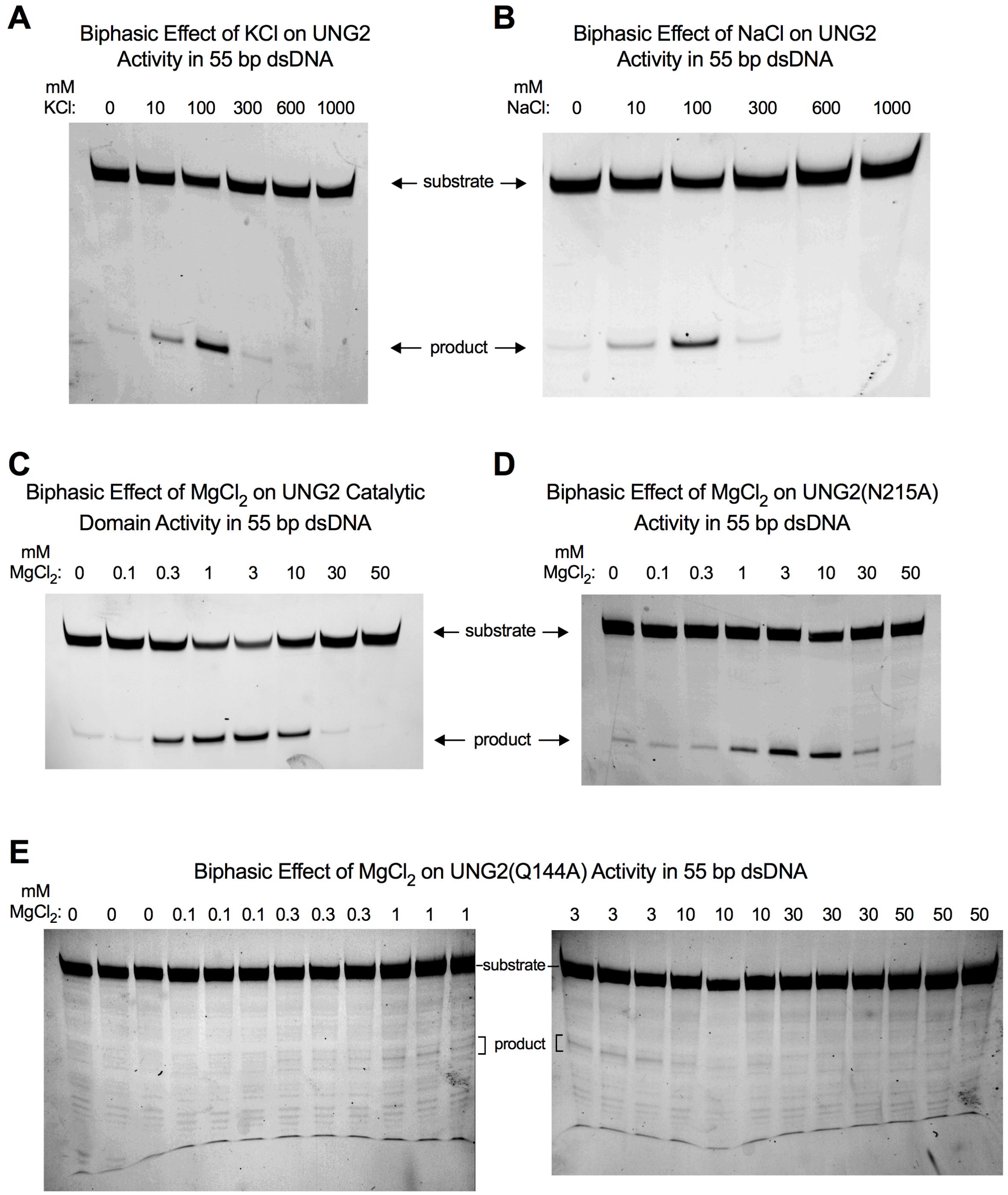

Figure S4. Atomistic molecular dynamics simulation of the UNG2 catalytic domain bound to a Mg^{2+} ion. (A) Initial structure of the simulation which was the protein and a coordinated Mg^{2+} ion. The atomic coordinates were taken directly from PDB code 5AYR. (B) Root mean square deviation (RMSD) of protein backbone α carbons throughout the trajectory referenced to the initial structure. (C) Close up view showing coordination of the Mg^{2+} ion by UNG2 side chains in the initial structure and in the final frame of the 3 μs simulation. (D) Distance between amino acid side chains from UNG2 and the Mg^{2+} ion that they stably coordinated throughout the trajectory. Specifically, the oxygen atoms on the side chains were responsible for coordinating the bound Mg^{2+}, especially from Asp145 and Asn215.

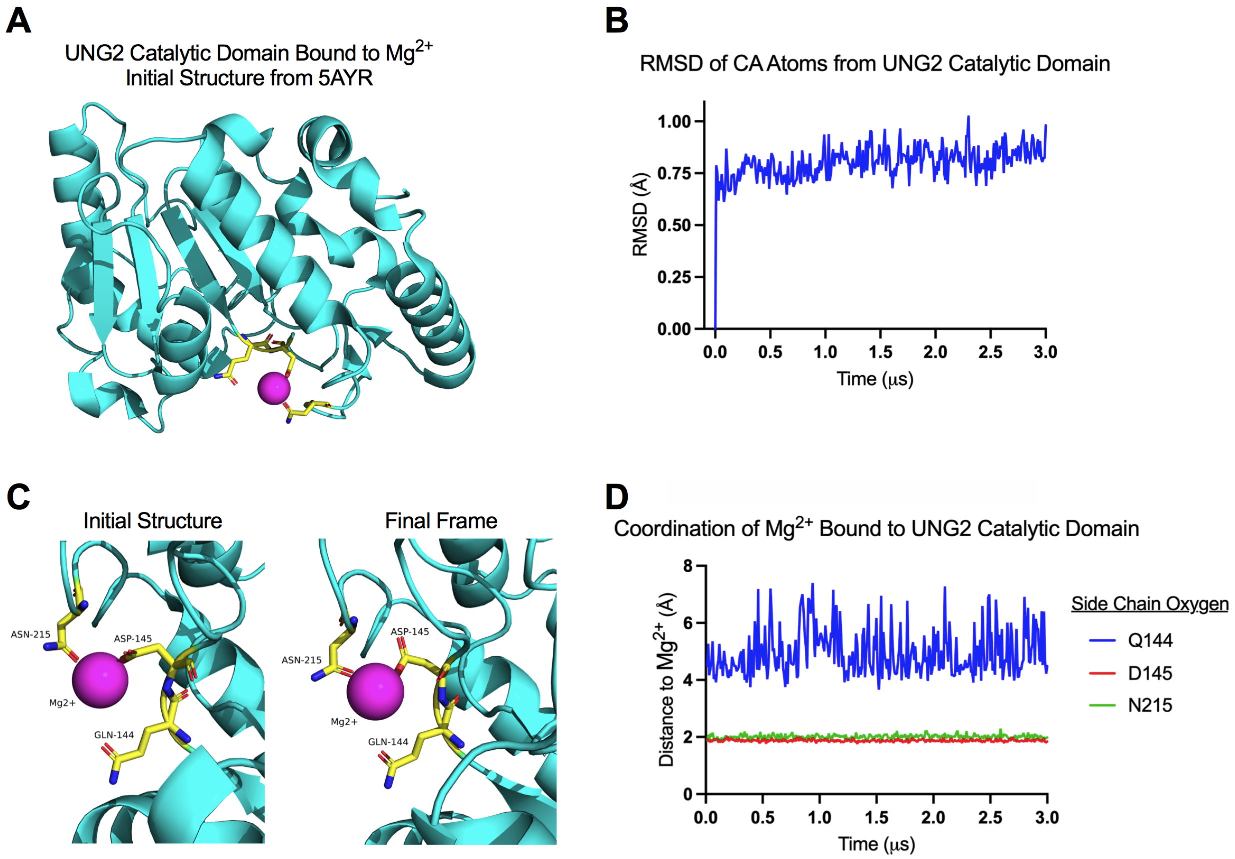

Figure S5. Relationship between the uracil excision activity of UNG2 in the absence of salt (parameter *d*) and the length of uracilated ssDNA substrate.

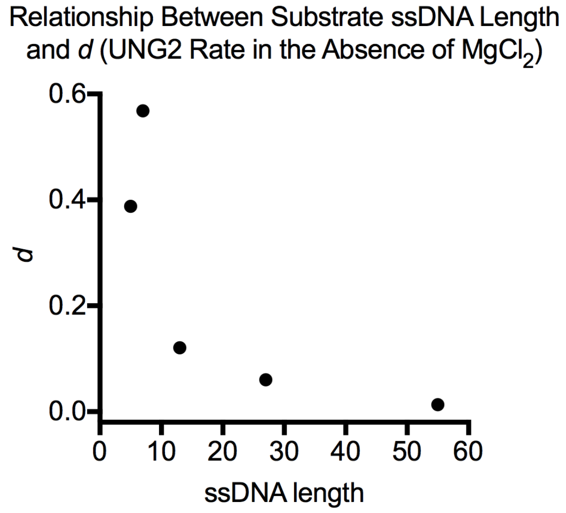

Figure S6. Transient, diverse structural states observed in atomistic molecular dynamics simulations of 13 nt and 7 nt uracil-containing ssDNA in the presence of Mg^{2+}. (A) Each panel shows the DNA backbone from the most common structural state observed throughout 300 ns simulations of a 13 nt oligo (separate simulations are number 1, 2, and 3). The structures were extracted from the simulations using a clustering algorithm that had a root mean square deviation cutoff of 2.5 Å. The percent of each simulation yielding the observed structural cluster was also shown on the bottom of each panel. Generally, even the most frequent structural states (shown below) were very transient and short-lived, reflected by the low percentage of time that the oligos assumed the structures. (B) The DNA backbone from the most common structural state observed throughout a 1 μs simulation of a 7 nt oligo is shown with the percent of the simulation yielding the observed structural cluster.

A

13 nt oligo

1 **2** **3**

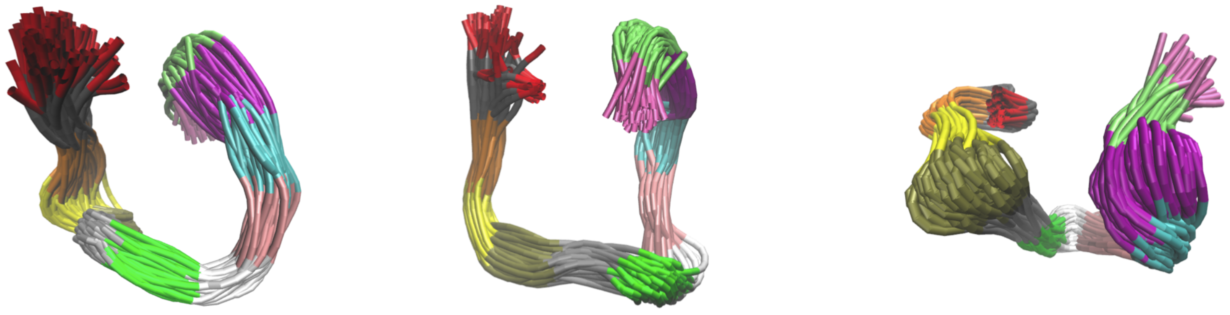

5.9% **2.7%** **7.4%**

B

7 nt oligo

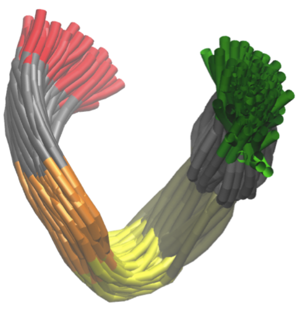

2.9%

Appendix.

Methods for Modeling UNG2 Activity in $MgCl_2$ Buffers using the Cedergreen Equation

The full datasets that we modeled can be found on zenodo (https://zenodo.org/records/14796319). The activity of the enzyme was described as the response value y for a given concentration of $MgCl_2$, which was the dose variable x. SAS Studio OnDemand for Academics webserver was used to fit curves to the data using the Cedergreen model of hormesis

$$y = c + \frac{(d-c) + f * \exp\left(\frac{-1}{x^{a}}\right)}{1 + \exp\left(b * ln\left(\frac{x}{e}\right)\right)} \qquad \text{(eq. A1)}$$

where d was the response of the enzyme in the absence of $MgCl_2$, c was the response of the enzyme in the presence of infinite doses of $MgCl_2$, a influences the rate of increase prior to the hormetic peak, b influences the rate of decrease after the hormetic peak, f was the hormesis parameter which is greater than zero in this type of hormetic curve, and parameter e sets a lower bound on the ED_{50}.[3] We assumed that the enzyme would be inactive in the presence of near infinite concentrations of $MgCl_2$ and fixed parameter c to 0 in all modeling. Methods and code for fitting curves with the Cedergreen model were previously released.[4] Parameters ED_{50}, M, LDS, y_{max}, and $y_{max\%}$ were determined with their appropriate equations,[5] and parameter values determined from eq. A1 were fixed during the calculation of these other variables.[3] As discussed below in *Select Modeling Notes*, other parameter values were sometimes constrained and selected in an iterative manner to achieve a satisfactory fit using eq. A1. R^2 and $R^2_{adjusted}$ values were calculated with Posit Cloud (RStudio) as previously described.[4]

Select Modeling Notes

(*i*) During graphing and modeling, the experimental data for the 0 mM $MgCl_2$ condition (no magnesium control) was changed to 0.001 mM $MgCl_2$ so it could be plotted as a value on the log_{10}-transformed *x* axis; 0.001 mM $MgCl_2$ was 100-fold lower than the lowest concentration actually tested (0.1 mM).

(*ii*) Parameter *a* was fixed to 1.5 when modeling the activity of UNG2 on 27 nt ssDNA; we omit this parameter value when considering correlations of curve parameters because of its uncertainty. A satisfactory fit for the activity of UNG2 on the 27 nt ssDNA could be determined by SAS without fixing parameter *a*, but the curve was unusually steep prior to the hormetic peak ($a > 3$) and the profile of the curve differed from others seen in this work. In reality, fixing *a* had minimal effect on the other parameters in eq. A1 or the goodness of fit (R^2 and $R^2_{adjusted}$) and mainly reduced the steepness of the hormetic increase, so we considered all other parameters from the 27 nt ssDNA curve valid. Other fixed values of *a* between 1 and 2 were also considered during modeling. Parameter *d* was also fixed when modeling the activity of UNG2 on 27 nt ssDNA; the value of 0.0546 was the mean k_{obs} (s^{-1}) for UNG2 processing the substrate in the absence of $MgCl_2$.

(*iii*) When modeling the activity of UNG2(N215A) on dsDNA, parameters *d* and *f* were fixed. The value of 0.0047 for parameter *d* was the mean k_{obs} (s^{-1}) for UNG2 processing the substrate in the absence of $MgCl_2$; fixing this parameter did not significantly alter the shape of the curve. However, without fixing parameter *f*, the curve fit by SAS had an unusually steep hormetic peak ($a > 3$), and the curve clearly underestimated y_{max} and did not fit the data at the hormetic peak. Without constraint, parameter *f* was determined by SAS to be 0.1444 compared

to the *f* value of 0.21 that we selected in an iterative manner. The greater *f* value in this case promoted a higher hormetic peak that more accurately estimated y_{max}.

(*iv*) In the absence of $MgCl_2$, the activity of UNG2(Q144A) on dsDNA was completely undetectable on our gels in triplicate assays (response = 0). Therefore, standard deviation could not be calculated for response values of 0, 0, and 0. Because the SAS code required error in the dataset in the form of 1/standard deviation (or "weight"), we set the weight to an arbitrarily high value for that dataset (200000). Even though we determined parameters for *d* and y_{max} (see below), we do not report a $y_{max\%}$ value for UNG2(Q144A) on dsDNA because it is exceptionally high and prone to error due to the small size of *d*.

With the appropriate parameterizations in SAS,[4,5] we were unable to determine ED_{50}, *LDS*, *M*, or y_{max} for the activity of UNG2(Q144A) on dsDNA. Parameters ED_{50} and *LDS* may be problematic to calculate because of the small range between *d* and *c*. To determine *M* and y_{max} for this dataset, we used python code written by ChatGPT version 4o (Python Code 1). Given the Cedergreen model (eq. A1) and parameter values determined from modeling (*a*, *b*, *c*, *d*, *e*, and *f*), the prompt was to determine the maximum *y* value and corresponding *x* from the curve.

Python Code 1. Determine parameters *M* and y_{max} given the Cedergreen equation and parameter values.

```
import numpy as np

# Define constants
a = 0.8674
b = 11.6036
c = 0
d = 0.000018
e = 9.0887
f = 0.04282

# Define the function based on the given equation
```

```
def y_function(x):
    numerator = d + f * np.exp(-1 / x**a)
    denominator = 1 + np.exp(b * np.log(x / e))
    return numerator / denominator

# Generate x values to evaluate the function
x_values = np.linspace(0.01, 100, 1000)

# Calculate y values for the x range
y_values = y_function(x_values)

# Find the maximum y value and the corresponding x value
y_max = np.max(y_values)
x_at_y_max = x_values[np.argmax(y_values)]

# Display the maximum y value and corresponding x value, rounded
to four decimal places
print("Maximum y value on the curve:", round(y_max, 4))
print("x value at maximum y:", round(x_at_y_max, 4))
```

Results of Hormetic Modeling for UNG2 / $MgCl_2$ Datasets

The activity of UNG2 and its variants on dsDNA substrates (k_{obs} values) in the presence of different $MgCl_2$ concentrations are shown in Figure S7. Curves were fit to the data with the Cedergreen model (eq. A1) and full parameters for the curves are shown in Table S3.

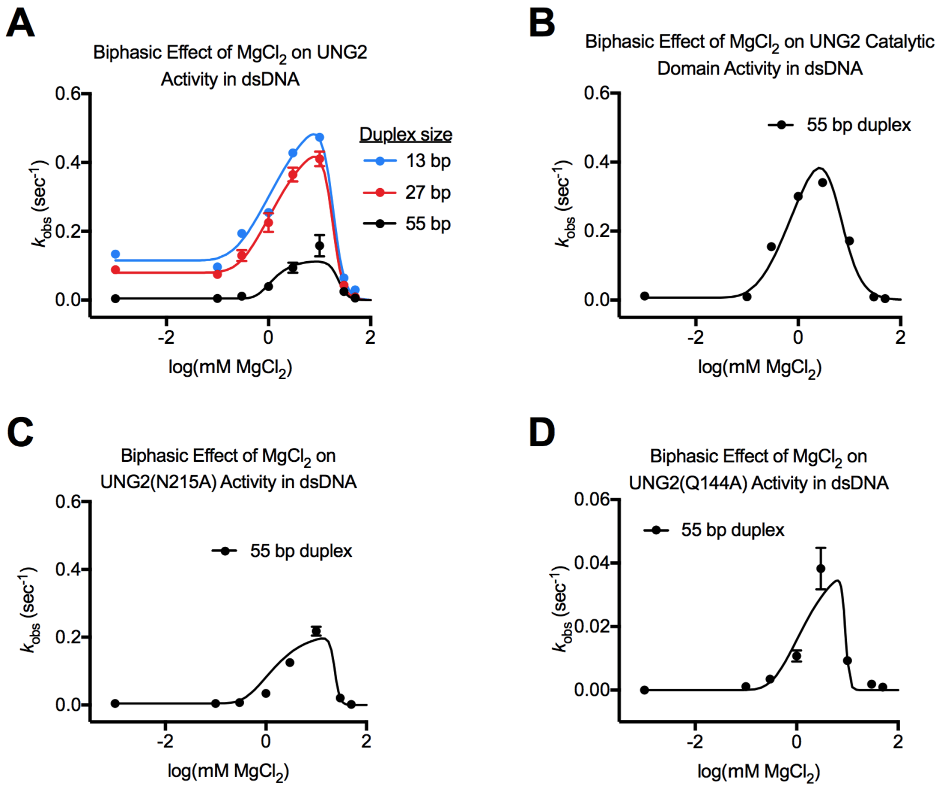

Figure S7. Hormetic dose-response curves showing the biphasic effects of $MgCl_2$ on the uracil excision activity of UNG2. For all assays, the buffer was 10 mM Tris-Cl and 0.1 mM EDTA (pH 8.0) with the indicated amount of $MgCl_2$ added to the buffer. The UNG2 concentration was between 1.5 and 4 nM, and the dsDNA substrate was 0.5 μM. The dsDNA substrate had a single uracil base near the center of duplex in the form of a U/A base pair.

Table S3. Parameters from Modeling the Activity of UNG2 on dsDNA Substrates of Different Lengths

	UNG2			Catalytic domain	UNG2(N215A)	UNG2(Q144A)
**Parameter**	**13 bp**	**27 bp**	**55 bp**	**55 bp**	**55 bp**	**55 bp**
a	0.6406	0.7439	1.6777	0.5840	0.9594	0.8674
b	3.9011	4.3175	4.7814	2.1709	8.7811	11.6036
c	0[a]	0[a]	0[a]	0[a]	0[a]	0[a]
d	0.1153	0.0799	0.0051	0.0069	0.0047[a]	0.000018
e	18.0927	17.7716	23.477	5.8005	23.3987	9.0887
f	0.5033	0.4333	0.1110	0.7857	0.21[a]	0.0428
ED_{50}	31.6165	30.9914	51.9313	68.0326	44.0676	ND
M	7.6841	7.9455	8.7440	2.6182	13.6336	6.3157
LDS	17.9455	18.6274	24.0284	48.9193	40.6952	ND
y_{max}	0.4821	0.4169	0.1122	0.3831	0.1517	0.0345
$y_{max\%}$	418.15	521.72	2204.28	5533.77	3228.03	ND
R^2	0.9709	0.9329	0.6720	0.9602	0.9040	0.8305
$R^2_{adjusted}$	0.9697	0.9315	0.6663	0.9584	0.8996	0.8228

[a]Parameter was fixed.

Additionally, the activity of UNG2 on ssDNA substrates (k_{obs} values) in the presence of different $MgCl_2$ concentrations are shown in Figure S8. Again, curves were fit to the data with the Cedergreen model (eq. A1) and full parameters for the curves are shown in Table S4.

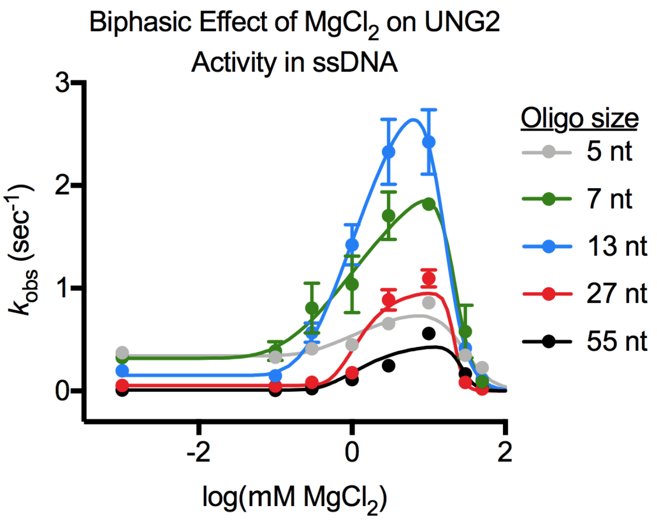

Figure S8. Hormetic dose-response curves showing the biphasic effects of $MgCl_2$ on the uracil excision activity of UNG2. For all assays, the buffer was 10 mM Tris-Cl, pH 8.0, and 0.1 mM EDTA with the indicated amount of $MgCl_2$ added to the buffer. The UNG2 concentration was between 0.75 - 3 nM, and the ssDNA substrate was 0.5 μM. The ssDNA substrate had a single uracil base near the center of the strand.

Table S4. Parameters from Modeling the Activity of UNG2 on ssDNA Substrates of Different Lengths

Parameter	5 nt	7 nt	13 nt	27 nt	55 nt
a	0.7140	0.4863	0.6840	1.5[a]	1.0431
b	2.3506	3.6662	2.9863	6.9821	4.9106
c	0[a]	0[a]	0[a]	0[a]	0[a]
d	0.3420	0.3183	0.1518	0.0546[a]	0.0086[a]
e	28.0470	21.6131	15.6667	21.5560	26.9411
f	0.5352	2.2650	3.5351	0.9298	0.4592

ED_{50}	50.2883	43.6292	55.9213	36.5585	87.2347
M	7.2972	8.9594	6.2928	11.4350	11.6550
LDS	32.7612	19.9758	43.8330	26.4574	32.7451
**y_{max}**	0.7312	1.8503	2.6393	0.9519	0.4267
**$y_{max\%}$**	213.81	581.30	1738.69	1744.00	4943.92
**R^2**	0.8615	0.7065	0.8688	0.9156	0.8704
**$R^2_{adjusted}$**	0.8552	0.6983	0.8654	0.9118	0.8645

[a]Parameter was fixed.

Modeling Methods and Results for UNG2 Activity in NaCl and KCl Buffers

Na^+ and K^+ ions affect UNG2 activity in a hormetic manner that is similar to Mg^{2+} ions, except that they are less potent (i.e., the hormetic dose-response curves are right-shifted). These monovalent cations likely affect UNG2 through the same mechanism as Mg^{2+}, except they interact less strongly with the DNA and require greater concentrations. Despite extensive efforts using the Cedergreen model (eq. A1), we were unable to fit satisfactory curves to our biphasic datasets showing the effects of NaCl or KCl on the enzymatic activity of UNG2. This was determined after numerous attempts with SAS where we constrained different variables, adjusted starting parameters, and artificially changed the weight or error of different datapoints.[4] We made similar efforts to model the datasets using the Cedergreen equation in python using code generated by ChatGPT. In fact, the SAS software could provide converged estimates for parameters of the Cedergreen model, but the curves vastly underestimated the steepness of the ascending slope (Figure S9, solid lines). We do not have an explanation for why the datasets using $MgCl_2$ could be satisfactorily modeled, but not the datasets using NaCl or KCl, considering

their similarities. All of the datasets had similar values for parameters *d* (~0.01) and *c* (0), the hormetic peaks ascended for ~1-2 orders of magnitude, and the descent from the hormetic peak was similarly steep.

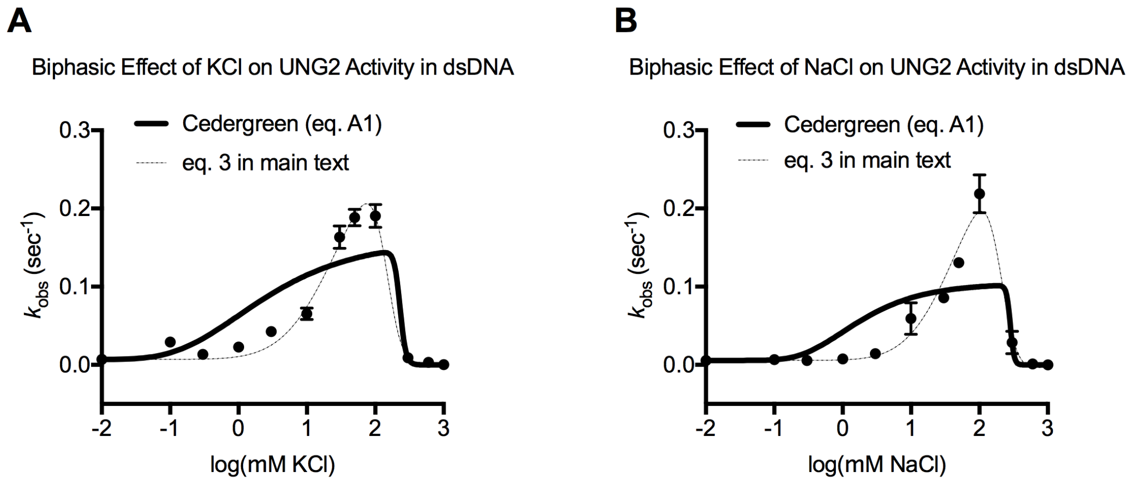

Figure S9. Hormetic dose-response curves showing the biphasic effects of KCl (panel A) and NaCl (panel B) on the uracil excision activity of UNG2. (A) The solid black line shows the converged curve fit to the data using eq. A1, whereas the dotted line shows the converged curve fit to the data using eq. 3 from the main text. Parameters for the solid black line are: a = 0.4624, b = 10.266, c = 0 (fixed), d = 0.00702 (fixed), e = 229.97, and f = 0.1522. (B) The solid black line shows the converged curve fit to the data using eq. A1, whereas the dotted line shows the converged curve fit to the data using eq. 3 from the main text. Parameters for the solid black line are: a = 0.6812, b = 15.9039, c = 0 (fixed), d = 0.0057 (fixed), e = 282.7, and f = 0.09845.

Supporting Information References

(1) Paradis, N. J.; Clark, A.; Gogoj, H.; Lakernick, P. M.; Vaden, T. D.; Wu, C. To Probe the Binding of TMPyP4 to C-MYC G-Quadruplex with in Water and in Imidazolium-Based Ionic Liquids Using Spectroscopy Coupled with Molecular Dynamics Simulations. *Journal of Molecular Liquids* **2022**, *365*, 120097. https://doi.org/10.1016/j.molliq.2022.120097.

(2) Paradis, N. J.; Clark, A.; Dutta, A.; Gogoj, H.; Vaden, T. D.; Wu, C. Elucidating the Stabilization Mechanism of a K+-Depleted c-MYC DNA G-Quadruplex in Hydrophobic Imidazolium-Based Ionic Liquids Using Spectroscopy Coupled with Molecular Dynamics Simulations. *Journal of Molecular Liquids* **2024**, *399*, 124407. https://doi.org/10.1016/j.molliq.2024.124407.

(3) Cedergreen, N.; Ritz, C.; Streibig, J. C. Improved Empirical Models Describing Hormesis. *Environ Toxicol Chem* **2005**, *24* (12), 3166–3172. https://doi.org/10.1897/05-014r.1.

(4) Abbaraju, V. D.; Robinson, T. L.; Weiser, B. P. Modeling Biphasic, Non-Sigmoidal Dose-Response Relationships: Comparison of Brain-Cousens and Cedergreen Models for a Biochemical Dataset. arXiv August 16, 2023. https://doi.org/10.48550/arXiv.2308.08618.

(5) Belz, R. G.; Piepho, H.-P. Modeling Effective Dosages in Hormetic Dose-Response Studies. *PLoS One* **2012**, *7* (3), e33432. https://doi.org/10.1371/journal.pone.0033432.
